# Phylogenetic Lineages of PRRSV-2 from Canada Reveal Patterns of Transboundary Spread and Two Novel Sub-Lineages in North America

**DOI:** 10.3390/pathogens15040346

**Published:** 2026-03-24

**Authors:** João P. Herrera da Silva, Igor A. D. Paploski, Robert Charette, Luc Dufresne, Sylvain Messier, Julie Bolduc, Mariana Kikuti, Nakarin Pamornchainavakul, Cesar A. Corzo, Kimberly VanderWaal

**Affiliations:** 1 Department of Veterinary Population Medicine, College of Veterinary Medicine, University of Minnesota, St. Paul, MN 55108, USAmkikuti@umn.edu (M.K.); corzo@umn.edu (C.A.C.); 2 Demeter Services Vétérinaires Inc., Lévis, QC G7A 3S8, Canadajbolduc@demetersv.com (J.B.)

**Keywords:** molecular epidemiology, phylogeography, transboundary, viral evolution, PRRSV-2, *Arteriviridae*

## Abstract

PRRSV-2 represents a major threat to the swine industry. Canada is one of the world’s leading pork producers and a major trading partner of live pigs with the United States, yet PRRSV-2 evolutionary dynamics in these two countries are often studied independently, partly due to limited publicly available sequence data from Canada. We analyzed more than 3000 PRRSV-2 ORF5 sequences collected between 2000 and 2024 from five Canadian provinces. Thirteen previously described sub-lineages were detected in Canada, while approximately one-third of the sequences could not be assigned to any known sub-lineage. Phylogenetic analyses incorporating global reference sequences revealed that most unclassified sequences clustered into four distinct monophyletic clades, exhibiting genetic distances greater than 9.5% from recognized sub-lineages. We propose two new sub-lineages, 1K and 1L, corresponding to clades that were prevalent and persistent over time, whereas the remaining two clades were rare and last detected in 2021. We reconstructed cross-border transmission histories and found that sub-lineages 1C, 1H, 1I, 1K, and 1L originated in Canada, whereas 1A, 1B, 1E, and 1F originated in the United States. Transmission patterns varied across sub-lineages, ranging from unidirectional to bidirectional movement. Our findings refine PRRSV-2 classification and provide insights to inform targeted surveillance, particularly at national borders.

## 1. Introduction

Since its emergence in the late 1980s [1], Porcine reproductive and respiratory syndrome virus type 2 (PRRSV-2) has caused substantial losses to the swine industry globally. The virus is endemic in Canada, where annual losses are estimated at approximately $150 million dollars annually [2]. Canada is the seventh-largest pork producer in the world, accounting for 2% of global pork production with an estimated total of 2.1 million tons produced between 2024 and 2025 [3]. The Canadian swine herd is estimated at 13.9 million animals in 2025, distributed across 7160 farms nationwide. Canada also plays a major role in the international swine trade. According to FAO data, in 2023 the country exported $3.1 billion in swine products and imported $3.9 billion [4]. Around 65% of the pork imported by the United States (USA) comes from Canada. In addition, the USA also imports live animals, with approximately 6.4 million pigs imported annually from Canada to the USA [5], underscoring the potential for cross-border dissemination of PRRSV-2 through animal movement and trade.

*Betaarterivirus americense* (Porcine Reproductive and Respiratory Syndrome Virus 2, PRRSV-2) belongs to the family Arteriviridae and is an enveloped, positive-sense single-stranded RNA virus. It has a 15 kb genome containing 10 open reading frames (ORFs). ORF1a and ORF1b encode genes involved in replication [6,7]. The 3′ portion of the genome encodes eight structural proteins, including five glycoproteins (GP2, GP3, GP4, GP5, GP5a), envelope (E), matrix (M) and nucleocapsid (N). PRRSV infects macrophage and dendritic cells, affecting the regulation of the inflammatory response and the induction of the adaptive immune response [8]. The main consequences of PRRSV-2 infection include reproductive losses, abortions, the birth of weak piglets, and respiratory disease [9].

PRRSV-2 exhibits remarkably high genetic diversity, driven by elevated substitution rates and frequent recombination. Substitution rates in regions encoding structural proteins range from 1.6 × 10^−3^ to 4.7 × 10^−2^ substitutions per site per year [10,11,12], placing PRRSV-2 among positive-sense RNA viruses with the highest reported substitution rates [13]. This accelerated molecular evolution contributes to the emergence of novel genetic variants, sometimes associated with perceived distinct phenotypic traits. Accurate classification of this genetic diversity is crucial for effective surveillance, epidemiological investigations, and the implementation of targeted biosecurity strategies [14]. Based on genetic distance and phylogenetic relationships of the ORF5 gene, PRRSV-2 has been classified into lineages, sub-lineages and variants [14,15,16,17,18,19]. To date, eleven lineages (L1–L11) have been formally described, which are further divided into 21 sub-lineages (e.g., 1A, 1B, 1C, etc.) [14,15,16,19]. In the USA context, sub-lineages are further divided into genetic variants for more fine-scale epidemiological tracking. From 2015 to 2025, 205 variants have been documented (PRRSLoom Variants, accessed September 23, 2025). The average genetic distance between lineages ranges from 9.1% to 17.2% [19]. Within lineages, the pairwise genetic distance between sub-lineage ranges from 6.9% to 14.7% [19]. Within sub-lineages, the average genetic distances between variants is ~5% (IQR 2.5–5.6%) [18].

Previous studies have reported that lineages 1 (L1), 2 (L2) and 5 through 9 (L5–L9) have circulated in Canada [20,21,22]. Sequence analyses of samples collected between 1998 and 2016 showed that L1 was prevalent in Quebec and Ontario, while most sequences from Manitoba were vaccine-like, belonging to L5 and L8 [21,22]. Non-vaccine sequences were rarely detected in Manitoba, although only a limited number of sequences from this province were analyzed in these studies [21,22]. Evidence points to Canada as a source of PRRSV-2 to USA; it has been suggested that PRRSV-2 L1 first emerged in Canada and was later imported into the USA [20,21]. Pamornchainavakul et al. [20] provided a more recent overview of PRRSV-2 spatiotemporal dynamics in North America, providing further evidence that the L1 viruses circulating in the USA emerged in Canada and additionally providing evidence that certain L1 sub-lineages moved back from the USA to Canada. However, the number of Canadian sequences analyzed was limited to fewer than one hundred [20]. The limited number of publicly available PRRSV-2 sequences from Canada prevents us from obtaining a comprehensive view of the genetic diversity of PRRSV-2 in the country. More recently, Lambert et al. explored the spatiotemporal dynamics of PRRSV-2 in Quebec, describing the presence of 38 genetic clusters, some of which were widely dispersed across the province and persisted for long periods, while others were more restricted to specific areas [23]. However, the placement of these clusters within the context of PRRSV-2 lineage and sub-lineage classification was based on lineages established using sequences from before 2008 [14,24], which differs from the updated lineage classification system established by Paploski et al. and Yim-im et al. [16,19] that accounts for the more recent diversification of the virus (particularly lineage 1, which is predominant in the USA) [15,16].

A current and comprehensive overview of PRRSV diversity in Canada is presented here. A total of 3000 ORF5 sequences were analyzed and classified using the global lineage classification system, allowing us to characterize the composition of Canadian viral populations. In addition, we mapped the spatial–temporal distribution of Canadian sub-lineages over a 24-year period (2000–2024). Finally, to gain insight into cross-border transmission patterns, we employed coalescent-based phylogeographic models to reconstruct the dispersal trajectories of sub-lineages shared between Canada and the United States. We focused our analysis of dispersal pathways on these two North American countries, as the United States and Canada are major trade partners.

## 2. Materials and Methods

### 2.1. Data Source

A total of 3573 PRRSV-2 ORF5 sequences from Canada were provided by Demeter Services Vétérinaires Inc., Lévis, QC, Canada, which accounts for 20–30% of national swine production. These sequences span the years 2000 to 2024 and were sampled across five Canadian provinces: Quebec (*n* = 2678), Ontario (*n* = 563), Manitoba (*n* = 316), Alberta (*n* = 11), and Saskatchewan (*n* = 5). To contextualize the diversity of PRRSV-2 circulating in Canada on a global scale, we analyzed the Canadian sequences alongside 78,496 ORF5 sequences representing worldwide diversity: 39,980 from GenBank (1990–2023, representing global diversity) and 35,162 from the Morrison Swine Health Monitoring Project (MSHMP) (1998–2024, representing USA diversity) [25,26]. Sequences lacking information on date or location (*n* = 13,986) or containing ambiguities or premature stop codons (*n* = 1365) were excluded from the analysis.

### 2.2. Sequence Classification, Estimation of Genetic Distances, and Sequences Filtering

The sequences were classified to lineage, sub-lineage and variant using the PRRS-Loom webtool [17]. Genetic distances among PRRSV-2 sub-lineages were calculated using the Sequence Demarcation Tool (SDT v1.2) [27], and expressed as the complement of the pairwise nucleotide identity (i.e., 100 − pairwise identity percentage). As our analyses were designed to focus on wild-type PRRSV-2, sequences with ≥95% identity to commercial vaccine strains were excluded [19]. For this purpose, ORF5 sequences assigned to vaccine-associated sub-lineages (5A, 7, 8A, and 8C) were compared with vaccine reference strains (Prevacent PRRS, KU131568; Ingelvac PRRSV MLV, AF066183.4; Prime Pac PRRSV RR, DQ779791.1; Ingelvac PRRSV ATP, DQ988080.1; and Fostera PRRSV, AF494042). Following the application of all filters, a total of 42,808 ORF5 sequences were retained for analysis, comprising 2361 from Canada, 13,110 from GenBank, and 27,337 from the MSHMP (Supplementary Figure S1). This dataset was divided into lineage-specific sets for further analysis.

### 2.3. Dataset Down-Sampling

Many of the analyses performed in this study are computationally intensive. To reduce computational burden, we adopted the spatiotemporal stratified subsampling methodology tested by Pamornchainavakul et al. [20]. This approach aims to ensure representativeness in terms of geographic region, sampling time, and genetic diversity (Supplementary Table S7 and Figures S1 and S3–S8). To account for potential biases introduced by subsampling and to ensure that our estimates were not artifacts of the subsampling procedure, we repeated the subsampling process five times, generating five distinct datasets per lineage (Supplementary Figures S1 and S3–S8), each of which were used to perform the subsequent analyses [20]. All sub-samplings were performed with replacement. Sub-sampling was conducted using TreeTrimmer [28], which utilizes a phylogenetic tree to estimate genetic distances based on branch lengths. Below, we detail each of the subsets constructed for specific analyses. Of note, subsampling was applied only to the global datasets and to datasets originating from the United States. Canadian sequences were retained without subsampling, except for the phylogeographic analyses of sub-lineage 1H, due to the large number of sequences available for this lineage. The proportional representation of Quebec and Ontario were relatively equal at the sub-lineage level (Supplementary Figures S3–S8).

#### 2.3.1. Down-Sampling for Recombination Analysis

Evidence of recombination that may have occurred in the Canadian sequences was investigated at different levels: within sub-lineages and between sub-lineages. To infer evidence of recombination within sub-lineages, the dataset was stratified by sub-lineage, and subsampling was performed as described above. Each resulting subset was then scanned for evidence of recombination (see Recombination Analysis). To reduce computational cost, the number of sequences in each sub-lineage-specific subset was limited to 600 (Supplementary Figure S1). For inter-lineage recombination investigation, we assembled a dataset in which at least 15 representative sequences from each sub-lineage circulating in Canada were present. In addition, at least 15 sequences were selected from each of the 21 recognized sub-lineages (Supplementary Figure S2C). The sequences were selected based on their geographic and temporal distribution, as well as their representation of the genetic diversity within each sub-lineage. The subsampling procedure was repeated five times, and each individual subset was subsequently screened for recombination.

#### 2.3.2. Down-Sampling for Phylogeographic Analysis

To infer the geographic origin and reconstruct the spread pathways of each sub-lineage, the dataset was first stratified by sub-lineage. Subsampling was then applied to USA sequences following the strategy described above to ensure representation of genetic diversity as well as spatial and temporal coverage. We targeted datasets of approximately 500 sequences per sub-lineage, generating five independent datasets for each sub-lineage (Supplementary Figures S1 and S3–S8). Sequences from Canada were retained in full whenever possible to preserve the available information from this region, as their numbers were generally much smaller relative to those from the USA. An exception was sub-lineage 1H, for which more than 1000 sequences were available from Canada alone, requiring subsampling to meet the dataset size threshold. For the remaining sub-lineages, Canadian sequences never exceeded 50% of the total dataset used in the phylogeographic analyses and were therefore included without subsampling. These datasets were subsequently used to reconstruct the spatio-temporal trajectories of each sub-lineage (see Phylogeographic Analysis).

#### 2.3.3. Down-Sampling for Estimation of Divergence Times

To estimate the divergence time of the ORF5 gene for the two newly identified sub-lineages and the two undetermined Canadian clades, we employed the same subsampling strategy described in the previous section. We selected sequences from each sub-lineage belonging to lineage 1, along with four additional novel Canadian clades (Supplementary Figure S2D). Our subsampling aimed to capture genetic diversity, as well as geographical and temporal representativeness.

### 2.4. Phylogenetic Analysis

We constructed a comprehensive phylogenetic tree comprising 6399 sequences representing the global diversity of PRRSV-2 (Supplementary Figure S2B). All 2361 wild-type sequences from Canada, were included in their entirety, while the remaining global sequences were subsampled using TreeTrimmer [28]. These additional sequences were drawn from a global dataset that included GenBank and MSHMP data. Subsampling was guided by representativeness in terms of genetic diversity, as well as geographic and temporal distribution. The phylogenetic trees were reconstructed using RAxML-NG [29] with the nucleotide substitution model determined by ModelTest-NG based on the Akaike Information Criterion [29]. The resulting phylogenetic trees were annotated with sub-lineage assignments generated by the PRRS-Loom classification webtool, as described in the previous section. Tree annotation was carried out using Microreact [30], enabling visualization of each sub-lineage’s taxonomic position and resolving the phylogenetic placement of Canadian sequences that were not assigned to any previously described sub-lineage, i.e., returned as “undetermined” by PRRS-Loom. Finally, after confirming the monophyly of the Canadian undetermined clades within the comprehensive global dataset, a phylogenetic tree was reconstructed containing only reference sequences (anchors) defined by Paploski et al. 2019 [16] and Yim-im et al. 2023 [19], along with the anchor sequences selected for the new sub-lineages established in this study. Fifty sequences from each clade representing proposed sub-lineages were selected using TreeTrimmer, prioritizing representativeness of each group’s genetic diversity. Because the objective of this analysis was to determine the phylogenetic placement of the detected clades relative to previously described sub-lineages, recombinant sequences were retained during phylogenetic reconstruction. Recombinant sequences were removed only for analyses aimed at estimating evolutionary and phylogeographic parameters.

### 2.5. Recombination Analysis

Down-sampled alignments for each sub-lineage (to assess intra-lineage recombination) and the multi-lineage dataset (inter-lineage recombination) were scanned for recombination using seven different methods: Rdp [31], Geneconv [32], Bootscan [33], Maximum χ2 [34], Chimaera [35], Siscan [36] and 3Seq [37], all implemented in the RDP5 program [38] using default parameters. Recombination events were considered reliable only when statistically supported by at least four different methods with *p*-values below 0.05.

### 2.6. Phylogeographic Analysis

Phylogeographic analyses were conducted after the removal of sequences with evidence of recombination. Sequences in the USA were attributed to tree geographic regions (defined by the Swine Health Information Center (SHIC) based on the USA swine density): Midwest (defined by SHIC as Region 3), Southwest (Region 2), and Eastern USA (Regions 4 and 5 combined) [39]. We focused our investigation on these regions because they represent the areas with the greatest data availability and the highest relevance for swine production. Regions 4 and 5 were analyzed jointly, owing to the limited number of sequences available from Region 4, which reflects the comparatively lower swine production in this area. For Canadian sequences, we employed official geopolitical demarcations, with regions corresponding to provinces. The analyses were conducted individually for each of the five down-sampled subsets (see above) for each sub-lineage. Phylogenetic trees were reconstructed using RAxML-NG [29] with the nucleotide substitution model determined by ModelTest-NG based on the Akaike Information Criterion [29]. Evidence for temporal signal was evaluated through root-to-tip regression on ML phylogenetic trees using TempeEst (Supplementary Table S5) [40]. To infer sub-lineage origin, i.e., the location of the most recent common ancestor, and to determine patterns of spread, we utilized discrete-space phylogeographic models using BEAST v1.10.4 [41]. We employed the GTR+I+G substitution model, which was determined using ModelTest-NG [29]. Both a strict molecular clock and an uncorrelated log-normal relaxed clock were evaluated. Marginal likelihoods for the competing clock models were estimated using the generalized stepping-stone sampling (GSS) method. Model selection was performed using log10 Bayes factors calculated from the differences in marginal likelihoods between the competing models, with the uncorrelated log-normal relaxed clock identified as the best-fitting molecular clock model (Supplementary Table S6) [42,43]. We used a non-reversible CTMC for the asymmetric discrete trait substitution model [44]. The coalescent model applied was the Bayesian Skygrid’s Gaussian Markov Random Field (GMRF) [45]. The analyses were performed with a Markov chain Monte Carlo (MCMC) chain length of 300 iterations. Each analysis was run three independent times to ensure consistency across runs. Log files from the independent runs were subsequently combined using LogCombiner. Convergence was evaluated using Tracer based on the effective sample size (ESS > 200) and the degree of independence between samples [41]. The phylogenetic trees were summarized as a Maximum Clade Credibility (MCC) tree in TreeAnnotator, discarding the initial 10% of the chains as burn-in [46]. Based on the results of the discrete space model, we estimated the number of transitions between pairs of regions using the relative transition rates and the BSSVS indicators. For each pair of regions, the number of transitions was calculated as the median of the relative transition rates, weighted by the mean tree length and the average substitution rate. As the analyses were performed in five subsampled datasets, the final number of transitions was estimated based on the average across the five runs (Supplementary Figures S14–S18).

### 2.7. Estimation of Divergence Times

To estimate the time to the most recent common ancestor (tMRCA) and the nucleotide substitution rate, we used the Bayesian Markov chain Monte Carlo (MCMC) method implemented in BEAST v1.4.10 [41]. The nucleotide substitution model (GTR + I + G) was selected using ModelTest-NG [29], and an uncorrelated lognormal relaxed molecular clock was applied based on log10 Bayes Factor comparison [42]. The analyses were run for 3 × 10^8^ MCMC iterations. Convergence was assessed in Tracer based on effective sample sizes (ESS > 200) and the degree of independence between samples. Phylogenetic trees were summarized using the Maximum Clade Credibility (MCC) tree in TreeAnnotator, with the first 10% of trees discarded as burn-in [46].

## 3. Results

### 3.1. Assignment of Sub-Lineages and Variants

**Figure 1:**
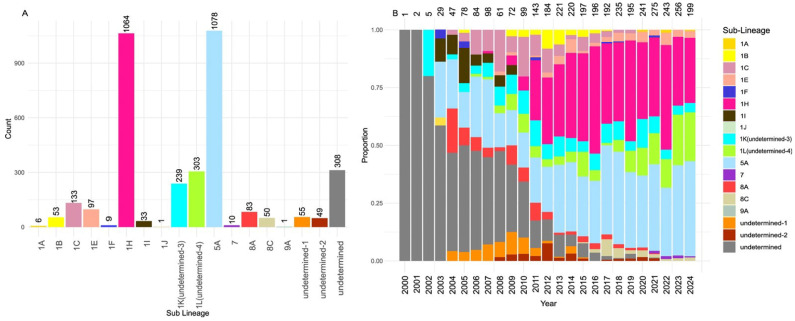
Number of Canadian sequences assigned to different sub-lineages (**A**) and their respective proportions per year (**B**). The numbers above the bars indicate the number of sequences assigned to each specific sub-lineage.

A total of 3573 ORF5 sequences collected between 2000 and 2024 from five Canadian provinces were analyzed. The distribution of sequences was as follows: Alberta (*n* = 11), Manitoba (*n* = 316), Ontario (*n* = 563), Quebec (*n* = 2678), and Saskatchewan (*n* = 5). The median annual number of sequences per year was 143 (interquartile range [IQR]: 72–220); during the first five years, the annual number of sequences remained below 50 (Figure 1B). For context, according to the Canadian Department of Agriculture, the swine population in 2023 was estimated at approximately 13.9 million animals, with 81% concentrated in three provinces: Quebec (31%), Ontario (26%), and Manitoba (24%) [3].

In total, we identified five lineages (1, 5, 7, 8 and 9), subdivided into 13 sub-lineages (1A, 1B, 1C, 1E, 1F, 1H, 1I, 1J, 5A, 7, 8A, 8C and 9A) (Figure 1A). A substantial number of the analyzed sequences (*n* = 954) were “undetermined” at the lineage level, representing 26.7% of the Canadian sequence set. Due to their high divergence from the lineage reference sequences used by PRRS-Loom, the model was unable to assign them to any known sub-lineage. At the genetic variant level, only 8 previously described variants (1C.23, 1E.1, 1F.1, 1I.1, 5A.1, 7.1, 8A.1 and 8C.1) were identified in the Canadian dataset, with a large portion of the sequences labeled as unclassified variants. Interestingly, some sequences for which sub-lineage could not be determined were classified as variant 1C.23 (89). The 1C.23 variant appears to be highly diverged from other sequences within sub-lineage 1C.

### 3.2. Phylogenetic Analyses and Sequence Comparisons Warrant Two New PRRSV-2 Sub-Lineages

After filtering out vaccine-like sequences (*n* = 1211, representing 33.89% of the dataset), a total of 2361 wild-type PRRSV-2 sequences from Canada were retained. Of these, 954 could not be assigned to any recognized sub-lineage. To better understand the relationship of sequences with undetermined lineage (*n* = 954) with recognized lineages, we constructed an ORF5 phylogenetic tree incorporating Canadian sequences alongside representative sequences from the 11 previously described lineages [19]. For the dataset representing these 11 lineages (21 sub-lineages), we included the reference sequences described by Paploski et al. (2021) [15] and Yim-im et al. (2023) [19], henceforth referred to as anchor sequences. In addition to the anchor sequences, for each lineage, a subset of ORF5 sequences available in GenBank and MSHMP was also included to obtain a more comprehensive representation of the genetic diversity of the 11 sub-lineages. In total, 6399 sequences were used to reconstruct the phylogenetic tree (Figure S10).

**Figure 2:**
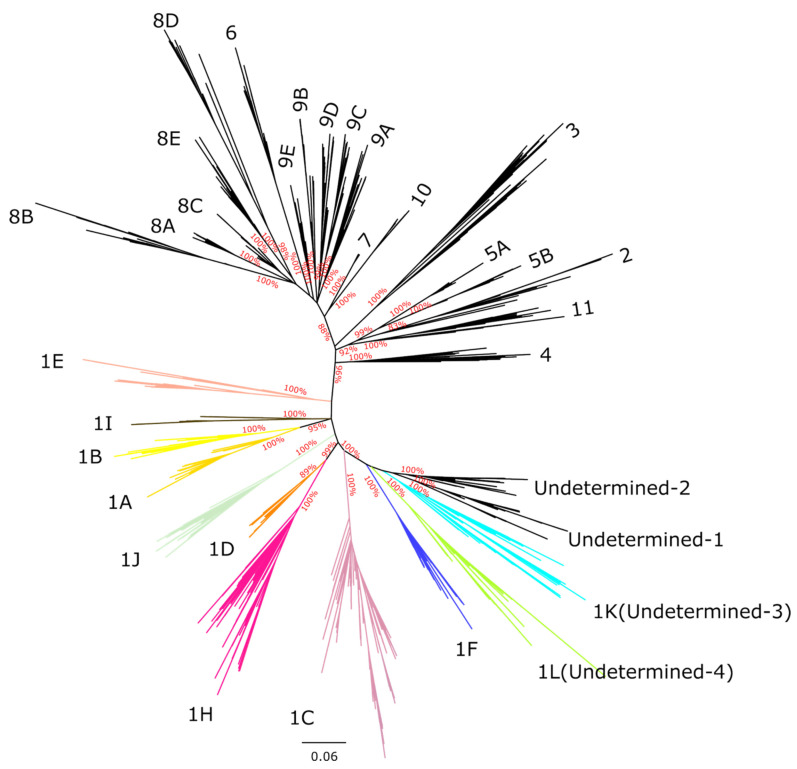
Unrooted maximum-likelihood phylogenetic tree based on ORF5 anchor sequences representing all sub-lineages globally, including newly identified Canadian sub-lineages and undetermined clades 1 and 2. The tree was constructed using RAxML-NG, employed the GTR + I + G4 substitution model. Numbers on the branches indicate branch support values for the basal nodes of each sub-lineage. The scale bar at the bottom represents the number of substitutions per site.

The majority of the described sub-lineages formed highly supported monophyletic clades (Supplementary Figure S10). Interestingly, the 1C and 1H sub-lineages from Canada clustered into separate monophyletic branches from other sequences of the same sub-lineages, suggesting that Canadian 1H and 1C sequences are genetically distinct from the 1C and 1H viruses circulating in the rest of the world (Supplementary Figure S10B). This likely indicates that 1C and 1H circulating in Canada are undergoing local differentiation with minimal viral exchange with other countries. 954 Canadian sequences were undetermined at the lineage level, and 646 of these clustered into four monophyletic clades with strong branch support (bootstrap > 99), here referred to as Undetermined 1 (55 sequences), Undetermined 2 (49 sequences), Undetermined 3 (239 sequences), and Undetermined 4 (303 sequences). This suggests that these sequences may represent previously undescribed sub-lineages. The four undetermined clades clustered near sub-lineage 1F, indicating they possibly share a common ancestor with this group (Figure 2 and Supplementary Figure S10).

To further investigate whether the four undetermined monophyletic clades should be allocated to new sub-lineages, we performed sequence comparisons to estimate the genetic distance between the four undetermined clades and all 21 previously described sub-lineages. These comparisons were performed with all available sequences, without employing any subsampling.

**Figure 3:**
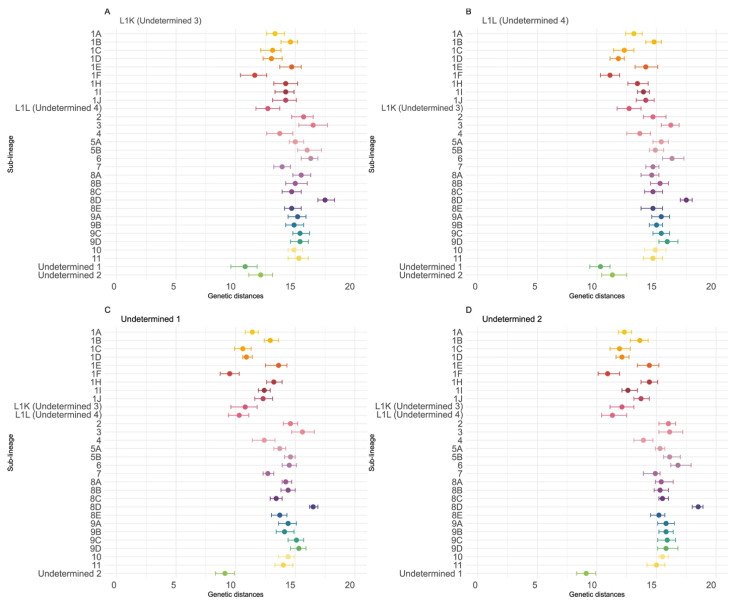
Genetic distances between newly identified Canadian lineages, undetermined Canadian clades, and all PRRSV-2 sub-lineages. (**A**–**D**) Genetic distances for 1K, 1L, undetermined clade 1, and undetermined clade 2 relative to all previously described PRRSV-2 sub-lineages and clades identified in this study. Dots represent the mean genetic distance, and bars indicate the interquartile range. Nucleotide identity percentages were estimated using SDT v1.2.

Undetermined-4 was most closely related to Undetermined-1 (mean genetic distance: 10.3%, IQR: 9.4–11.1), followed by 1F (11.1%, IQR: 10.3–11.9) and Undetermined-2 (11.3%, IQR: 10.4–12.5); distances to all other sub-lineages exceeded 11.8% (Figure 3, Table S1). A similar pattern was observed for Undetermined-3, which also was most closely related to Undetermined-1 (10.8%, IQR: 9.6–11.8), followed by 1F (11.6%, IQR: 10.4–12.6) and Undetermined-4 (12.1%, IQR: 11.1–13.1), with all other distances >13% (Figure 3, Table S1). Consistent with these findings, Undetermined-1 was most closely related to Undetermined-2 (9.1%, IQR: 8.3–9.9), then 1F (9.5%, IQR: 8.7–10.3) and Undetermined-4 (10.3%, IQR: 9.4–11.1) (Figure 3, Table S1). Finally, Undetermined-2 mirrored this relationship, being most similar to Undetermined-1 (9.1%, IQR: 8.3–9.9), followed by 1F (10.9%, IQR: 10.1–11.9) and Undetermined-4 (11.3%, IQR: 10.4–12.5). Intra-clade distances further support their distinctiveness, with mean genetic distances of 6.5% (IQR: 4–9.4) for Undetermined-4, 10.4% (IQR: 7.6–12.1) for Undetermined-3, 5.3% (IQR: 3.8–6.5) for Undetermined-1, and 6.5% (IQR: 3.6–8.1) for Undetermined-2 (Table S1). These values reinforce that the four clades found in Canada are highly divergent from other sub-lineages while also exhibiting considerable internal diversity.

Based on the frequency of each undetermined clade, their persistence over time, and their genetic distinctiveness, we propose the establishment of two new PRRSV-2 sub-lineages within L1 to accommodate two of the four undetermined clades: sub-lineage 1K, comprising the 303 sequences in the Undetermined-3 clade, and sub-lineage 1L (239 sequences), comprising the Undetermined-4 clade. Undetermined clades 1 and 2 represent approximately 1.6% and 1.4% of the sampled sequences in Canada and were last identified in our dataset in 2015 and 2021, respectively (Figure 2). Therefore, we believe that the creation of new sub-lineages to classify rare clades that are no longer circulating is not justified. However, we should not rule out the possibility of the future re-emergence of these clades.

Interestingly, one of the two of the newly proposed sub-lineages, 1L, and the other two undetermined clades appear to circulate exclusively in Canada (Supplementary Figure S10B, Table S3). On the other hand, 1K has been detected previously in the USA (Supplementary Table S3), with those sequences placed in a divergent clade of sub-lineage 1C (variant 1C.23). These findings suggest that the classification of variant 1C.23 should be revised, with those sequences being reassigned to sub-lineage 1K, as new data reveals that it belongs to sub-lineage 1K rather than 1C.

As part of the establishment of the new sub-lineages, we selected 50 anchor sequences for sub-lineages 1K, 1L and 1H to represent the genetic diversity within each group (GenBank accession numbers PV179105 to PV179253, PV179365 to PV179414). Finally, we reconstructed a phylogenetic tree containing only the anchor sequences, representing the 21 previously described sub-lineages along with the two newly proposed sub-lineages, as well as the two remaining undetermined clades, Undetermined 1 and Undetermined 2. The anchor-based tree (Figure 2) mirrored the topology of the global PRRSV-2 phylogeny (Supplementary Figure S10).

### 3.3. Establishment of New PRRSV-2 Genetic Variants

To create a systematic and unified nomenclature across the USA and Canada for denoting genetic variants of PRRSV-2 below the sub-lineage level, the Canadian PRRSV-2 sequences were assigned to either existing or new variants following the criteria for genetic variants and naming conventions used in the USA [17,18]. These conventions have been widely adopted by diagnostic laboratories and monitoring programs in the USA and have proven useful in facilitating communication among producers, practitioners, and surveillance systems [17,18]. Briefly, variants are defined by identifying phylogenetic clusters in the tree consisting of at least 5 sequences with mean patristic distances < 7%, and with bootstrap values > 85 for the ancestral node in the phylogeny [17,18]. Using this criteria, two new variants within 1C were established, 1C.41 and 1C.42. Of the 97 Canadian sequences assigned to lineage 1E, only 20 were attributed to a previously described variant (variant 1E.1) found in the USA. To accommodate Canadian 1E sequences, variants 1E.10 through 1E.12 were created. In contrast, none of the 1H variants present within the USA were detected in Canada. Thus, for 1H, we established new variants 1H.36 to 1H.49 to cover the diversity found in Canada. For 1K, we created 1K.1 to 1K.4, which include the former 1C.23 variant, and finally, to accommodate 1L, variants 1L.1 to 1L.4 were created. The average genetic distance within sequences belonging to the same variant ranges 0.33 to 8.29% with a median of 3.31% (Supplementary Table S4), and the average distance from the most closely related variant ranges 5.63 to 10.91% (Supplementary Table S4). Some sequences remain unclassified due to uncertainties in properly positioning them within the classification because they represent poorly resolved phylogenetic subgroups (low bootstrap values) or due to a lack of closely related sequences in sufficient numbers to support the creation of new variants.

The sub-lineages and variants presented in this manuscript have been incorporated into the PRRS-Loom webtool so that end-users can determine the classifications of their own sequences (sub-lineages: https://stemma.shinyapps.io/PRRSLoom/; variants: https://stemma.shinyapps.io/PRRSLoom-variants/).

### 3.4. Evidence of Intra-Sub-Lineage Recombination Was Detected in a Small Number of 1K and 1L Sequences

We did not find evidence suggesting that sub-lineages 1K and 1L emerged as a result of recombination on the ORF5 gene. No inter-lineage recombination was detected in the Canadian PRRSV-2 population. Recombination signals were only found within sequences of the same sub-lineage, but only for a small number of sequences within sub-lineages 1K and 1L. Two recombination events were detected, one involving 27 sequences in 1L, and the other one in 1K, evidenced in only one sequence (Supplementary Table S2).

### 3.5. Differential Spatial Distribution of Sub-Lineages

Variation in the spatial distribution and abundance of PRRSV-2 sub-lineages was observed across Canadian provinces. A total of 13 distinct sub-lineages were identified in Quebec (1B, 1C, 1E, 1F, 1H, 1I, 1K, 1L, 5A, 7, 8A, 8C, and 9A), making it the province with the highest sub-lineage diversity. In Quebec, sub-lineage 1H was the most abundant (*n* = 908, representing 33.9% of Quebec sequences), followed by the vaccine-like sub-lineage 5A (*n* = 664, 24.8%), 1L (*n* = 304, 11.3%), and 1K (*n* = 141, 0.05%) (Supplementary Figure S9). Notably, sub-lineage 1L and the undetermined clades 1 and 2 were detected exclusively in Quebec.

In Ontario, eight distinct sub-lineages were identified in the province (1B, 1E, 1F, 1H, 1I, 1K, 5A, and 8A). 1H was also the most prevalent sub-lineage (*n* = 156, representing 27.7% of Ontario sequences), followed by 5A (*n* = 133, 23.6%), 1K (*n* = 97, 17.2%), and 1E (*n* = 89, 15.8%) (Supplementary Figure S9 and Table S3). In Manitoba, a total of four sub-lineages were detected. Among them, 1K was the only wild-type sub-lineage found in this province. The others, 5A, 8A, and 8C, were vaccine-related, showing less than 5% genetic divergence from vaccine strains, corroborating previous findings that PRRSV-2 strains circulating in Manitoba are predominantly closely related to vaccine strains [21,22]. In Alberta, sub-lineage 1A (*n* = 6) and vaccine-associated 5A (*n* = 5) were detected, while in Saskatchewan, all five sampled sequences belonged to sub-lineage 5A. Sub-lineage 1A was found exclusively in Alberta.

Spatial distributions should be interpreted with caution because the number of Canadian sequences analyzed per province did not necessarily mirror the distribution of the swine population across provinces, as some provinces are overrepresented relative to others. While Quebec accounts for 31% of the national swine herd, it contributed 74% of the sequences. Ontario, with 26% of the national swine herd, contributed 15% of sequences; Manitoba, representing 24% of the swine population, contributed 8%; Alberta accounts for 10% of the herd but contributed only 3% of the sequences; and Saskatchewan, which holds 8% of the swine population, accounted for just 1.3% of the sequences. This pattern suggests one of two possibilities: either PRRSV-2 prevalence outside Quebec is lower, or that our dataset has a more limited ability to capture sequences from regions outside Quebec. As a result, our capacity to assess viral diversity in regions beyond Quebec, especially during the first decade of the study, may be disproportional.

### 3.6. Quebec and the USA Midwest Are Potential Hubs for the Dispersal of PRRSV-2 Sub-Lineages Between Canada and the United States

Canada and the USA share a border and engage in animal trade [5], which serves as a pathway for the spread of PRRSV-2 between the two countries [20,21]. To investigate the geographic origins of ancestral populations of each wild-type sub-lineage and patterns of spread between the two countries, we conducted a discrete-space phylogeographic analysis, considering four Canadian provinces (Quebec, Ontario, Alberta, Manitoba) and three USA regions: the Midwest, the Eastern USA, and the Southwest. Other Canadian provinces and USA regions were not considered due to lack of data. For this analysis, only sub-lineages identified in these two countries were analyzed. Sub-lineages related to modified live vaccine strains were not analyzed, as these sub-lineages are repeatedly seeded to different areas through vaccine use [21].

**Figure 4:**
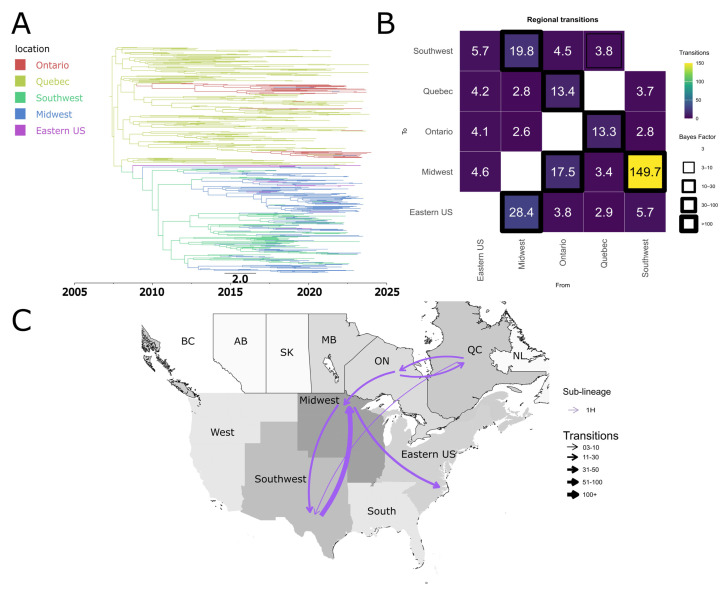
Spatiotemporal dynamics of the PRRSV-2 1H sub-lineage in North America. (**A**) The panel on the top left shows a maximum clade credibility (MCC) phylogenetic tree reconstructed using a discrete-space diffusion model. (**B**) The heatmap panel on the top right displays number of transition between different regions, represented by color gradients and numerical values within each cell. Warm colors indicate high transition counts, while cool colors indicate low transitions counts. The thickness of the black boxes around the cells indicates the magnitude of the Bayes factor; only Bayes factors above 3 were considered significant. (**C**) The bottom panel shows spread pathway. Arrows indicate the direction of spread, and their thickness represents the transition numbers. The USA regions shown correspond to swine-producing areas, defined based on animal density according to SHIC. The abbreviations on the Canada map refer to different provinces.

**Figure 5:**
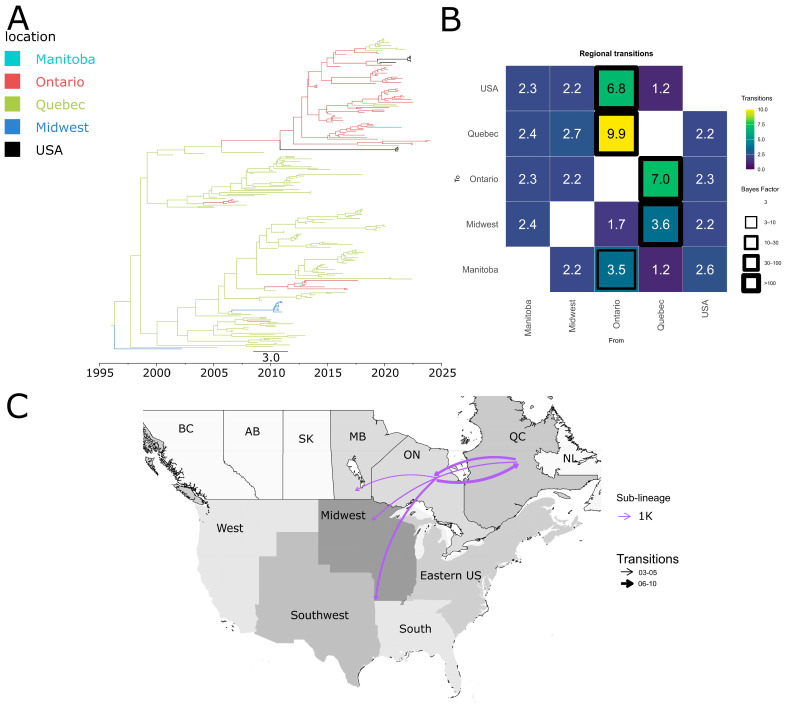
Spatiotemporal dynamics of the PRRSV-2 1K sub-lineage in North America. (**A**) The panel on the top left shows a maximum clade credibility (MCC) phylogenetic tree reconstructed using a discrete-space diffusion model. (**B**) The heatmap panel on the top right displays number of transition between different regions, represented by color gradients and numerical values within each cell. Warm colors indicate high transition counts, while cool colors indicate low transitions counts. The thickness of the black boxes around the cells indicates the magnitude of the Bayes factor; only Bayes factors above 3 were considered significant. (**C**) The bottom panel shows spread pathway. Arrows indicate the direction of spread, and their thickness represents the transition numbers. The USA regions shown correspond to swine-producing areas, defined based on animal density according to SHIC. The abbreviations on the Canada map refer to different provinces.

**Figure 6:**
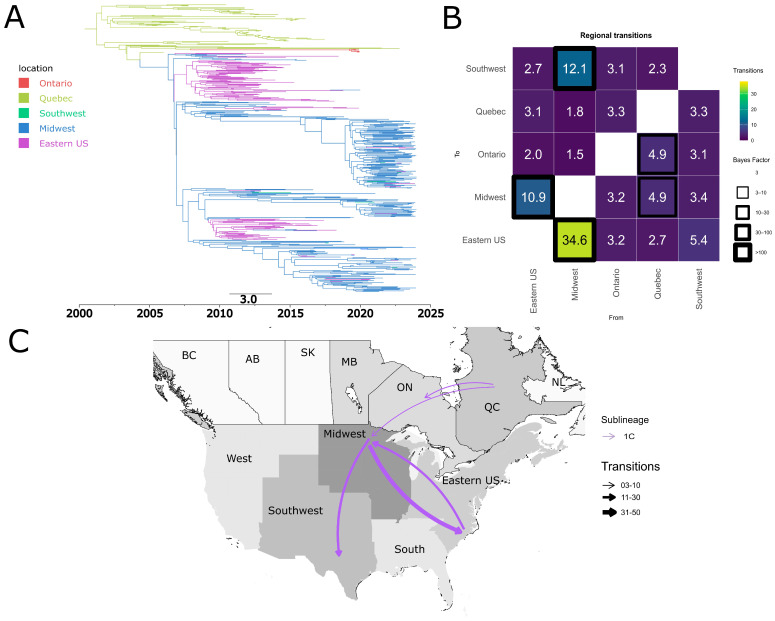
Spatiotemporal dynamics of the PRRSV-2 1C sub-lineage in North America. (**A**) The panel on the top left shows a maximum clade credibility (MCC) phylogenetic tree reconstructed using a discrete-space diffusion model. (**B**) The heatmap panel on the top right displays number of transition between different regions, represented by color gradients and numerical values within each cell. Warm colors indicate high transition counts, while cool colors indicate low transitions counts. The thickness of the black boxes around the cells indicates the magnitude of the Bayes factor; only Bayes factors above 3 were considered significant. (**C**) The bottom panel shows spread pathway. Arrows indicate the direction of spread, and their thickness represents the transition numbers. The USA regions shown correspond to swine-producing areas, defined based on animal density according to SHIC. The abbreviations on the Canada map refer to different provinces.

**Figure 7:**
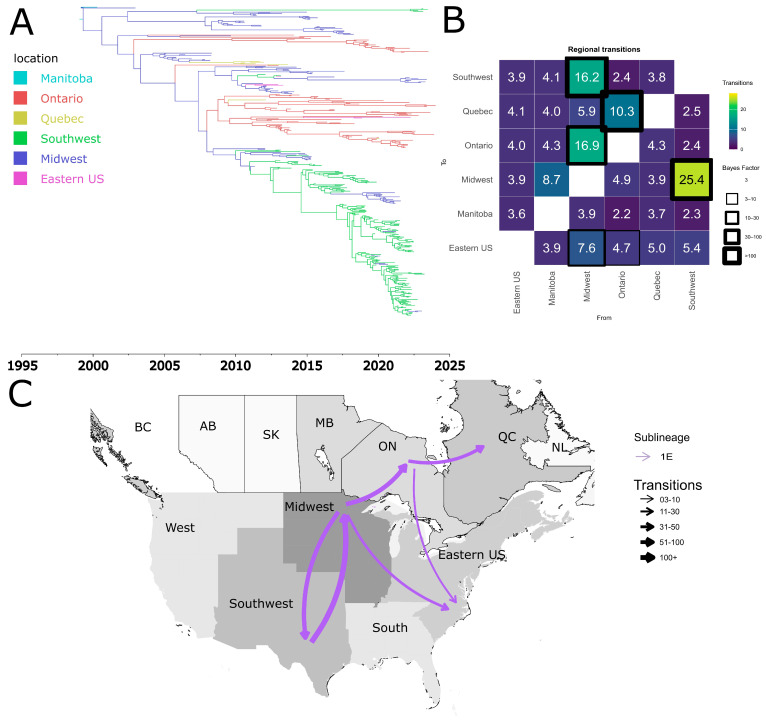
Spatiotemporal dynamics of the PRRSV-2 1E sub-lineage in North America. (**A**) The panel on the top left shows a maximum clade credibility (MCC) phylogenetic tree reconstructed using a discrete-space diffusion model. (**B**) The heatmap panel on the top right displays number of transition between different regions, represented by color gradients and numerical values within each cell. Warm colors indicate high transition counts, while cool colors indicate low transitions counts. The thickness of the black boxes around the cells indicates the magnitude of the Bayes factor; only Bayes factors above 3 were considered significant. (**C**) The bottom panel shows spread pathway. Arrows indicate the direction of spread, and their thickness represents the transition numbers. The USA regions shown correspond to swine-producing areas, defined based on animal density according to SHIC. The abbreviations on the Canada map refer to different provinces.

**Figure 8:**
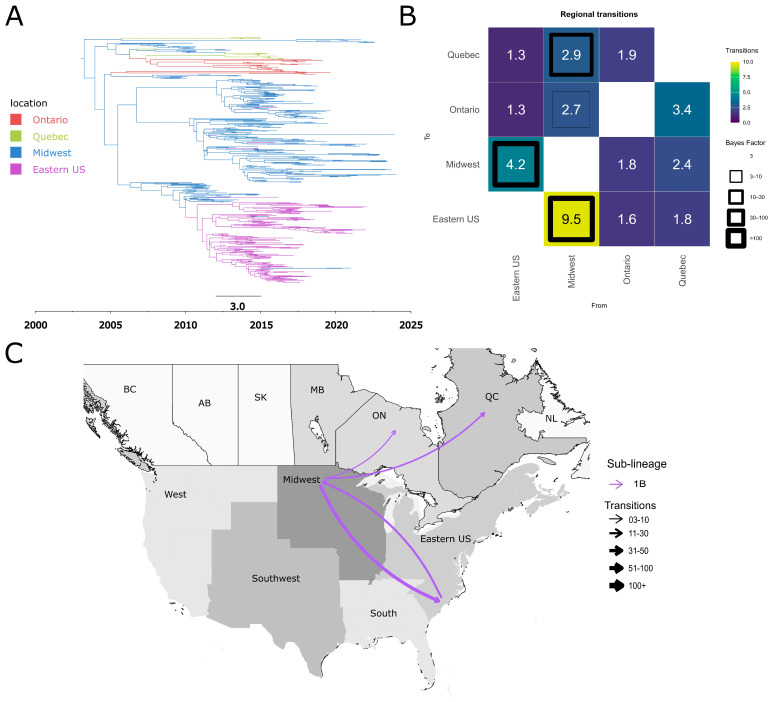
Spatiotemporal dynamics of the PRRSV-2 1B sub-lineage in North America. (**A**) The panel on the top left shows a maximum clade credibility (MCC) phylogenetic tree reconstructed using a discrete-space diffusion model. (**B**) The heatmap panel on the top right displays number of transition between different regions, represented by color gradients and numerical values within each cell. Warm colors indicate high transition counts, while cool colors indicate low transitions counts. The thickness of the black boxes around the cells indicates the magnitude of the Bayes factor; only Bayes factors above 3 were considered significant. (**C**) The bottom panel shows spread pathway. Arrows indicate the direction of spread, and their thickness represents the transition numbers. The USA regions shown correspond to swine-producing areas, defined based on animal density according to SHIC. The abbreviations on the Canada map refer to different provinces.

Our analysis indicates that the Canadian province of Quebec, along with the USA Midwest are key regions where certain sub-lineages originated. Specifically, sub-lineages 1C, 1H, 1I, 1K, 1L, as well as the undetermined clade 1 and clade 2, appear to have originated in Quebec (Figure 4, Figure 5, Figure 6, Figures S10 and S11). Sub-lineages 1A, 1B and 1E originated in the USA Midwest, consistent with previous findings (Figure 7, Figure 8 and Figure S13) [20], and were introduced to Canada. Sub-lineage 1L, as well as the undetermined clades 1 and 2, were restricted to Canada.

Our results indicate that the common ancestor of sub-lineage 1H viruses circulating in the USA and Canada was in Canada, specifically, in the province of Quebec (Figure 4A). 1H spread from Canada to the USA, with both Ontario and Quebec as source regions for introduction events into USA (Figure 4B,C). The earliest introduction event appears to have occurred before 2010 (Figure 4A). A total of ~3.7 transitions from Quebec and ~17.5 from Ontario to the Midwest were inferred (Figure 4B). At the intra-country level, we also observed bidirectional dispersion dynamics of 1H between Quebec and Ontario (Figure 4B,C).

Sub-lineage 1K emerged in Canada and subsequently spread to the USA Midwest (Figure 5C), with five introduction events apparent in the consensus phylogenetic tree (Figure 5A). However, because this is a consensus tree, some possible transition events identified in the analysis did not appear in the tree. In total, ~6.8 transition events between Ontario and the USA, and ~3.6 transitions between Quebec and Midwest were detected (Figure 5B). Most of these events occurred in the 2020s, though there was one introduction that occurred in the early 2010s (Figure 5A). However, 1K occurred at low frequencies in the USA, suggesting that it has yet to become established in the country. Some 1K sequences from the USA lacked precise regional information, making it difficult to determine their internal dispersion patterns within the country. Within Canada, bidirectional movements of 1K were observed between Quebec and Ontario, as well as dispersal from Ontario to Manitoba.

The dispersion patterns of 1C between Canada and the USA were unidirectional. 1C was introduced from Quebec to the Midwest of the USA (Figure 6B,C). Additionally, the dispersion of 1C from Quebec to Ontario was observed. Approximately 4.8 transitions were identified from Quebec to the Midwest, with the earliest transition likely occurring around 2004 (Figure 6A,B).

Sub-lineage 1E exhibited a complex dynamic with multiple introduction events from the USA into Canada (Figure 7). The Midwest and Eastern USA were identified as potential sources of dispersal from the United States to Canada (Figure 7B,C). Local dispersal events were also observed within Canada, specifically from the province of Ontario to Quebec (Figure 7B,C). Additionally, we found evidence of reintroductions from Canada into the United States from Quebec to the Eastern USA (Figure 7B,C). Our results indicate that the earliest introduction event of 1E in Canada occurred in the early 2000s in Ontario. All of the additional introduction events between the two countries appear to have taken place throughout the first two decades of the 2000s (Figure 7A).

Sub-lineage 1B also followed a unidirectional dispersal pattern between the two countries, having been introduced into Quebec and Ontario as separate introductions from the USA Midwest (Figure 8B,C). The absence of evidence for inter-provincial transmission within Canada suggests that these introduction events in Ontario and Quebec may have been independent (Figure 8B,C). Our inferences suggest that the introduction events likely occurred between 2005 and 2010 (Figure 8A).

The number of available 1I sequences was very limited, with only 46 sequences in total, all which date to before 2010. We reconstructed the spread pathway of sub-lineage 1I (Supplementary Figure S11), which exhibited a pattern of exportation and reintroduction between Canada and the USA, initially migrating from Quebec to the USA Midwest, and subsequently from the Midwest back to Ontario (Supplementary Figure S11B,C). The introduction events in the USA occurred in the late 1990s and early 2000s (Supplementary Figure S11A).

In contrast to previously reported findings [20], our analyses suggest that 1F originated in the Midwest region of the United States rather than in Canada and was introduced into Quebec (Supplementary Figure S12A). Other early work on Lineage 1 PRRS suggests a Canadian origin of older sub-lineages such as 1F [14]. However, we believe that this discrepancy may be because our analysis only included 1F sequences, whereas the previous work showing a Canadian origin of 1F included older unclassified lineage 1 sequences that were circulating in Canada and that may have been ancestrally related to 1F. From Quebec, 1F likely dispersed to Ontario (Supplementary Figure S12B,C).

Finally, we reconstructed the spread pathways for sub-lineage 1A. 1A exhibited a unidirectional dispersal pattern between countries, originating in the United States and being introduced into Canada only in Alberta, where it remained confined and was only detected by six sequences (Supplementary Figure S13B,C). The introduction of 1A in Alberta appears to have occurred between 2007 and 2010 (Supplementary Figure S13A).

### 3.7. Age of Sub-Lineages 1K, 1L, and Undetermined Clades

**Figure 9:**
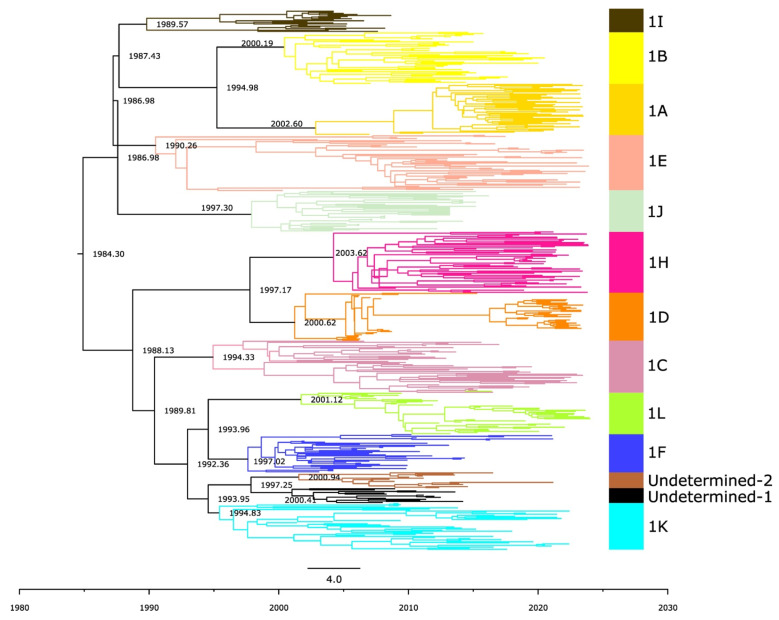
MCC phylogenetic tree of ORF5 from PRRSV-2 Lineage 1. Branch lengths represent the mean divergence time in years, which is also indicated in the node labels. The different colors represent each of the PRRSV-2 sub-lineages. The tree is automatically rooted using a relaxed log-normal molecular clock.

To determine the emergence time of new sub-lineages and undetermined clades, we reconstructed a single time-scaled phylogenetic tree with all of lineage 1 viruses (Figure 9). The estimated substitution rate per site per year was 9.63 × 10^−3^ (95% HPD: 8.77 × 10^−3^ to 1.10 × 10^−2^), which aligns with estimates reported in previous studies [10]. 1K emerged in Canada around 1995 (95% HPD: 1992–1996). 1L emerged in approximately 2001 (95% HPD: 2000–2004). The two undetermined clades, Undetermined 1 and Undetermined 2, emerged almost simultaneously, the first in ~2000 (95% HPD: 1998–2003) and the second in ~2001 (95% HPD: 1998–2004). These dates align well with the notion that most current sub-lineages resulted from evolutionary diversification in the 1990s [20].

## 4. Discussion

PRRSV-2 remains one of the major threats to the swine industry. Its rapid evolutionary dynamics, driven by high rates of mutation and recombination, continually drive the emergence of new genetic variants and pose significant challenges to control efforts [11,12,15,47,48,49,50]. Although substantial progress has been made toward classifying and organizing PRRSV-2 diversity [14,15,17,18,19], our understanding remains fragmented due to uneven regional sampling and the limited availability of genomic data in public repositories. To partially address this gap and expand our understanding of PRRSV-2 diversity, we analyzed more than 3000 ORF5 sequences from Canada. Our results reveal that Canada harbors distinct PRRSV-2 diversity. We also describe two new sub-lineages designated as 1K and 1L. Phylogeographic analyses indicate clear patterns of geographic structuring, suggesting that viral populations circulating in Canada are, to some extent, distinct from those in the United States, likely reflecting local diversification following introduction events. Moreover, our analyses corroborate previously reported emergence and dispersal patterns and provide further evidence that sub-lineages 1C and 1H emerged in Canada, while Lineages 1B, 1E, and 1A originated in the USA [20].

The identification of two new sub-lineages, 1K and 1L, advances our understanding of the global diversity of PRRSV-2 and highlights how much remains to be uncovered before a comprehensive picture of this pathogen’s evolution can be achieved. Sub-lineages 1K and 1L form well-supported, highly divergent monophyletic clades (>11% mean nucleotide divergence) that have circulated in Canadian herds for over a decade, yet had remained unrecognized in global classification schemes.

Given the high substitution rates observed for PRRSV-2, it is expected to observe clades displaying relatively broad levels of within-sub-lineage genetic variability [10,11,12]. Similar levels of intra-sub-lineage dissimilarity have also been reported for sub-lineages 1E and 1I. There is no explicit genetic distance cutoff for the demarcation of PRRSV-2 sub-lineages. In practice, classification has been based on phylogenetic clustering and genetic distance relative to reference sequences from previously described sub-lineages, although these values vary depending on the groups considered [14,15,16,17,18,19]. Our proposal for establishing these sub-lineages is supported by the strong monophyly of the identified clades, which remained stable when we reconstructed a more comprehensive phylogenetic tree using more than 6000 sequences sampled globally. In addition, the restriction, or near restriction, of these groups to Canada further supports this interpretation, suggesting that these lineages likely emerged and diversified locally within the country and have been circulating there for more than 20 years.

Additionally, we expanded the variant classification system to the sequences sampled in Canada. This system was recently implemented and is currently being applied for fine-scale classification of PRRSV-2 ORF-5 sequences in the USA [17]. Given the practical use of this system is for epidemiological monitoring, it allows a more precise tracking of clades and enables reconstruction of dispersal trajectories at a more granular level. However, some of the variants proposed here, such as 1K.2 and 1K.4, exhibit intra-variant genetic distances above ~7% (Table S4), which is larger than most genetic variants defined in the USA. The variant classification system is designed to be dynamic with established rules for splitting variants, hence the variants presented here represent provisional categories that are constantly reassessed updated as new data are incorporated [17]. The decision to consider such diverse groups as variants is largely driven by the practical aspects of surveillance and by the need to facilitate communication among producers.

Interestingly, in a previous study, Lambert et al. [22] characterized the diversity of PRRSV-2 in Canada, contrasting the composition of Canadian PRRSV-2 diversity with a subset of reference sequences representing lineages L1 through L9 as defined by Shi et al. [14], which described global diversity at that time (up to 2008). Lambert et al. [22] reported a distinct monophyletic clade within lineage L1 that appeared to be endemic to Canada, first detected as early as 2007 and continuously expanding thereafter [22]. Interestingly, the oldest sequences of 1K and 1L analyzed here date from the same period as this highly divergent L1 cluster described by Lambert et al. [22]. However, any correspondence between these genetic clusters and our newly proposed sub-lineages was not possible, as reference sequences from the clades described by Lambert et al. are not publicly available. In our study, we adopted the nomenclature and classification framework proposed by Paploski et al. and Yim-im et al. [15,19] to name these newly identified sub-lineages. This system has become widely used in the USA and internationally [51,52,53], promoting greater consistency in PRRSV-2 lineage definitions. Recognizing, naming, and standardizing PRRSV-2 lineages is more than a taxonomic exercise; it is a necessary step toward building a coherent global framework that enables clear communication among stakeholders, improves the interpretation of epidemiological connections, supports coordinated regional and international control efforts, and helps prevent the reintroduction or undetected spread of previously absent viral groups.

Our phylogeographic analyses revealed clear patterns of geographic structuring between PRRSV-2 populations from Canada and the USA, evidenced by distinct separations among viral populations from the two countries. In some cases, this structuring was particularly pronounced, as observed for sub-lineages 1H and 1C, which formed two well-supported monophyletic clades largely restricted to one country, suggesting infrequent viral exchange between Canada and the USA for these groups.

The restricted geographic distribution and infrequent cross-border movements observed for sub-lineages 1C and 1H can be readily explained by the timing of their emergence. Most of the introduction events detected between Canada and the USA occurred before the 2010s, when neither 1C and 1H had yet expanded substantially in either country. The expansion of 1C in the USA began toward the end of the first decade of the 2000s, whereas 1H only became widespread after 2010 in both countries [15,16,20]. A plausible explanation for the limited evidence of more recent introductions is that strengthened biosecurity measures related to animal movement may now play a key role in restricting cross-border transmission. However, we did not have access to detailed data to formally evaluate this hypothesis.

For the other sub-lineages, clustering by geographic location was still apparent, although not as clearly dichotomous as that observed for 1H and 1C. This pattern suggests comparatively higher levels of cross-border viral exchange and more occasional introductions between Canada and the United States than those inferred for 1H and 1C. Sub-lineage 1K exhibited evidence of multiple importation events from Canada into the United States, though it does not appear to have become well established there. In contrast, the phylogenies of sub-lineages 1B and 1E suggest more frequent bidirectional spread across the border. Sub-lineage 1A, although widespread in the United States, was detected only at very low frequency in Canada (*n* = 6) and exclusively in the province of Alberta. This lineage became prevalent in the USA around 2014 [16], and its limited presence in Canada may reflect sporadic and localized introduction events. Finally, our results suggest that sub-lineage 1F originated in the USA, in contrast to earlier reports suggesting a Canadian origin [20], introducing some uncertainty regarding its true geographic source. Precise conclusions regarding the movements of 1I and 1A between the two countries are constrained by the representativeness of the available dataset; 1I appears to have ceased circulating in both countries after 2010, with only a few sequences detected.

Overall, our findings support a scenario in which PRRSV-2 Lineage 1 was initially introduced into the United States through imports from Canada, subsequently diversified locally, and certain sub-lineages were later reintroduced into Canada, consistent with observations from previous studies [5]. Most introduction events from Canada to the United States appear to have occurred in the late 1990s and early 2000s, with the exception of more recent introductions of 1K during the 2020s, which may suggest failures in biosecurity protocols.

The USA imports approximately 6 million live pigs from Canada each year, whereas Canada imported a total of only 82,137 pigs from the USA over the entire 2000–2024 period [5]. Considering this asymmetry, it is reasonable to expect that introductions of PRRSV-2 from the United States into Canada via infected pigs are possible but likely occur at a much lower frequency than in the opposite direction. Additionally, this lower risk is particularly evident given that 41.8% of pigs imported into Canada during this period were breeding animals, which likely originated from high-biosecurity premises with active PRRSV monitoring [5]. In the early 2000s, there was a substantial increase in the trade of live pigs between the two countries, which is consistent with the timing of most PRRSV-2 sub-lineage exchanges inferred from our phylogeographic analyses [5].

In contrast, 73.3% of pigs exported from Canada to the United States over the past 24 years were feeder pigs weighing less than 50 kg, populations typically managed under lower biosecurity and biocontainment conditions after arrival in the U.S [5]. These movement patterns help explain the greater number of inferred lineage transitions from Canada to the United States observed in our phylogeographic analyses. Most Canadian imports from the USA occur through northern and central states, supporting our inference that the central USA represents a key source region for PRRSV-2 introductions into Canada.

Additional mechanisms of introduction may also contribute to cross-border transmission. Trucks transporting animals can act as mechanical vehicles for PRRSV-2 [54], and imported semen has been identified as another potential pathway [55], although boar studs are generally subject to strict biosecurity and continuous PRRSV surveillance. It is also possible that other uncharacterized routes play a role, underscoring the complexity of PRRSV-2 transmission dynamics across national borders.

Despite providing one of the most comprehensive assessments of PRRSV-2 diversity in Canada to date, our study has some limitations that should be acknowledged. Although our dataset encompasses the five provinces responsible for more than 97% of Canadian swine production, there is a sampling imbalance among regions. In particular, Manitoba represents approximately 24% of national production but accounts for only about 10% of the sequences in our dataset. In addition, other regions are also underrepresented, such as Alberta and Saskatchewan, which together account for nearly 18% of national production [3]. In Manitoba, we predominantly detected vaccine-like sequences and observed a low presence of wild-type strains. A genuine concern regarding the nature of our data may therefore be raised, as these sequences were generated through routine diagnostic submissions rather than active surveillance, which may inevitably introduce bias and result in an incomplete representation of the true viral diversity circulating in the province. On the other hand, our findings are consistent with previous studies reporting a low frequency of wild-type strain circulation in Manitoba [21,22].

This uneven representation may reflect genuinely lower PRRSV-2 prevalence outside Québec and Ontario, but it also likely stems from the origin of our dataset, which was largely derived from a major swine health clinic with fewer clients in other provinces, especially in earlier years. Consequently, early introduction and transition events may be underrepresented, limiting our ability to fully reconstruct the timing and geographic context of sub-lineage emergence.

Given the large volume of data used for phylogenetic inference, conducting full-scale analyses was computationally prohibitive, largely due to the limited scalability of BEAST for datasets of this size. Therefore, we subsampled our dataset, a process that inherently carries the risk of introducing bias if not carefully executed. To minimize such artifacts, subsampling was conducted at least five times for each dataset, with replacement, ensuring that the temporal and spatial diversity within each sub-lineage was adequately represented. Importantly, our subsampling strategy was not designed to equalize the number of sequences across regions or time periods, but rather to preserve the genetic diversity of the dataset across space and time (Supplementary Figures S3–S8). In the process, over-represented regions were down-sampled more extensively than other regions.

In Canada, there is a clear imbalance in sampling intensity among provinces, and the spatial distribution of sequences does not necessarily reflect the geographic distribution of the swine population. Uneven sampling could potentially influence phylogeographic inference, including the estimated location of the most recent common ancestor [56]. However, most analyses were conducted at the sub-lineage level, and the spatial imbalance among Canadian provinces was far less pronounced within sub-lineage datasets. An exception is sub-lineage 1H, for which most sequences originate from Quebec, though our sub-sampling strategy preferentially reduced these sequences. Furthermore, our results were consistent across all of five independent replicates and aligned with previous studies [20,21], supporting the robustness of the phylogeographic patterns observed here.

Nonetheless, we acknowledge that the true number of viral transitions between regions is likely higher than inferred, as temporal clustering and uneven sequencing intensity may have led to an underestimation of these events. Despite these limitations, the dataset presented here represents a substantial improvement over what was previously available in public repositories and provides a critical foundation for future research. Expanding genomic surveillance in under-sampled regions will be essential to refine our understanding of PRRSV-2 diversity, elucidate viral dispersal pathways, and strengthen coordinated regional and continental control strategies.

## 5. Conclusions

In this study, we expand the understanding of PRRSV-2 genetic diversity in North America. We propose the establishment of two new PRRSV-2 sub-lineages from Canada that likely emerged between the late 1990s and early 2000s. In addition, we identified two other highly divergent monophyletic clades related to these novel sub-lineages; however, these clades do not appear to be currently circulating in the country. Our phylogenetic analyses indicate that the PRRSV-2 sub-lineages circulating in Canada are genetically divergent from those found in the United States. To further organize this genetic diversity, we defined new PRRSV-2 variants, thereby expanding the recently implemented variant classification system [17]. Our findings suggest that Canada may be considered one of the centers of diversification for PRRSV, with the provinces of Quebec and Ontario, in particular, acting as sources for the dispersal of sub-lineages 1C, 1H, 1I, and 1K. Most sub-lineages exhibited a unidirectional dispersal pattern to or from the USA, except for sub-lineages 1E and 1I, which originated in the USA, were introduced into Canada, and later dispersed back into the USA. These results indicate that the PRRSV-2 dispersal route between Canada and the USA is largely unidirectional for most sub-lineages. These findings offer important insights for guiding targeted surveillance and biosecurity strategies aimed at preventing the cross-border introduction of PRRSV-2 variants not yet detected in either country.

## Data Availability

Portions of the dataset are privately owned by the production systems and may be subject to restrictions. However, the data can be made available upon reasonable request to the corresponding author, contingent upon permission from the respective production systems. Reference sequences for the newly established sub-lineages (anchor sequences for 1K and 1L), as well as for undetermined clades 1 and 2, are available in GenBank under accession numbers PV179104 to PV179253 and PV179270 to PV179414.

## Supplementary Materials

The following supporting information can be downloaded at: https://www.mdpi.com/article/10.3390/pathogens15040346/s1, Figure S1. Workflow of PRRSV-2 dataset processing and sub-lineage segregation. Sequences were retrieved from GenBank (*n* = 39,980), MSHMP (*n* = 35,162), and Canadian sequences generated in this study *(n* = 3573). After filtering GenBank sequences lacking collection date or geographic region (*n* = 13,110), USA sequences (*n* = 9501) and Canadian sequences (*n* = 118) were selected. Combined datasets from the USA and Canada were subsequently segregated by sub-lineage. For each major sub-lineage (1A, 1B, 1C, 1E, 1F, 1H, 1I, and 1K), subsampling was performed five times to generate representative datasets for phylogeographic (*n* = 500) and recombination (*n* = 600) analyses. The left and right panel shows the distribution of Canadian sequences across sub-lineages after segregation and subsampling. Figure S2. Workflow of sequence datasets and analyses. (A) Segregation of PRRSV-2 sequences from GenBank (*n* = 39,980), MSHMP (*n* = 35,162), and Canadian sequences from this study (*n* = 3354) based on sub-lineages for sequence comparison. (B) Construction of a phylogenetic tree using a combined dataset after filtering GenBank sequences lacking date or geographic region information (*n* = 13,110) and sub-sampling (*n* = 3045). (C) Assessment of inter–sub-lineage recombination using a combined dataset with sub-sampling (*n* = 20 per sub-lineage). (D) Estimation of divergence times using a combined dataset with sub-sampling (*n* = 500). Figure S3. (A) Original dataset of sub-lineage 1A sequences. (B–F) Sub-sampled datasets from five independent simulations showing the number of sequences per year across regions in the USA and Canada. Sub-sampling was performed to standardize sample size across years, geographic regions, and genetic distances. Figure S4. (A) Original dataset of sub-lineage 1C sequences. (B–F) Sub-sampled datasets from five independent simulations showing the number of sequences per year across regions in the USA and Canada. Sub-sampling was performed to standardize sample size across years, geographic regions, and genetic distances. Figure S5. (A) Original dataset of sub-lineage 1B sequences. (B–F) Sub-sampled datasets from five independent simulations showing the number of sequences per year across regions in the USA and Canada. Sub-sampling was performed to standardize sample size across years, geographic regions, and genetic distances. Figure S6. (A) Original dataset of sub-lineage 1E sequences. (B–F) Sub-sampled datasets from five independent simulations showing the number of sequences per year across regions in the USA and Canada. Sub-sampling was performed to standardize sample size across years, geographic regions, and genetic distances. Figure S7. (A) Original dataset of sub-lineage 1H sequences. (B–F) Sub-sampled datasets from five independent simulations showing the number of sequences per year across regions in the USA and Canada. Sub-sampling was performed to standardize sample size across years, geographic regions, and genetic distances. Figure S8. Temporal distribution of PRRSV-2 sequences in Canada for three original datasets. (A) Sub-lineage 1K, (B) sub-lineage 1F, and (C) sub-lineage 1I. Bars represent the number of sequences collected per year, stratified by geographic region (Eastern, Western, Ontario, Quebec, and Atlantic). Numbers above the bars indicate the count of sequences for each year–region. Figure S9. Proportion of PRRSV-2 ORF5 sequences per year by province. Each panel represents a Canadian province. The bars show the proportion of each sub-lineage over time within each province, with the number on top of each bar indicating the total number of sequences represented by that bar. The different colors represent the various sub-lineages. Figure S10. Unrooted phylogenetic trees reconstructed using maximum likelihood. The phylogenetic trees were generated using PRRSV-2 ORF-5 sequences from a dataset comprising over 6000 global sequences, representing the diversity within each PRRSV-2 sub-lineage. Both panels display the same phylogenetic tree. (A) Each color represents a different PRRSV-2 sub-lineage. (B) Each color represents the geographic origin of each sequence. The phylogenies were reconstructed using RAxML-NG with the GTR + I + G4 nucleotide substitution model. Figure S11. Spatiotemporal dynamics of the PRRSV-2 1I sub-lineage in North America. (A) The panel on the top left shows a maximum clade credibility (MCC) phylogenetic tree reconstructed using a discrete-space diffusion model. (B) The heatmap panel on the top right displays number of transition between different regions, represented by color gradients and numerical values within each cell. Warm colors indicate high transition counts, while cool colors indicate low transitions counts. The thickness of the black boxes around the cells indicates the magnitude of the Bayes factor; only Bayes factors above 3 were considered significant. (C) The bottom panel shows spread pathway. Arrows indicate the direction of spread, and their thickness represents the transition numbers. The USA regions shown correspond to swine-producing areas, defined based on animal density according to SHIC. The abbreviations on the Canada map refer to different provinces. Figure S12. Spatiotemporal dynamics of the PRRSV-2 1F sub-lineage in North America. (A) The panel on the top left shows a maximum clade credibility (MCC) phylogenetic tree reconstructed using a discrete-space diffusion model. (B) The heatmap panel on the top right displays number of transition between different regions, represented by color gradients and numerical values within each cell. Warm colors indicate high transition counts, while cool colors indicate low transitions counts. The thickness of the black boxes around the cells indicates the magnitude of the Bayes factor; only Bayes factors above 3 were considered significant. (C) The bottom panel shows spread pathway. Arrows indicate the direction of spread, and their thickness represents the transition numbers. The USA regions shown correspond to swine-producing areas, defined based on animal density according to SHIC. The abbreviations on the Canada map refer to different provinces. Figure S13. Spatiotemporal dynamics of the PRRSV-2 1A sub-lineage in North America. (A) The panel on the top left shows a maximum clade credibility (MCC) phylogenetic tree reconstructed using a discrete-space diffusion model. (B) The heatmap panel on the top right displays number of transition between different regions, represented by color gradients and numerical values within each cell. Warm colors indicate high transition counts, while cool colors indicate low transitions counts. The thickness of the black boxes around the cells indicates the magnitude of the Bayes factor; only Bayes factors above 3 were considered significant. (C) The bottom panel shows spread pathway. Arrows indicate the direction of spread, and their thickness represents the transition numbers. The USA regions shown correspond to swine-producing areas, defined based on animal density according to SHIC. The abbreviations on the Canada map refer to different provinces. Figure S14. Spatiotemporal dynamics of the PRRSV-2 1A sub-lineage in North America. (A–E) The heatmap panel on the top right displays number of transition between different regions, represented by color gradients and numerical values within each cell. Warm colors indicate high transition counts, while cool colors indicate low transitions counts. The thickness of the black boxes around the cells indicates the magnitude of the Bayes factor; only Bayes factors above 3 were considered significant. Figure S15. Spatiotemporal dynamics of the PRRSV-2 1B sub-lineage in North America. (A–E) The heatmap panel on the top right displays number of transition between different regions, represented by color gradients and numerical values within each cell. Warm colors indicate high transition counts, while cool colors indicate low transitions counts. The thickness of the black boxes around the cells indicates the magnitude of the Bayes factor; only Bayes factors above 3 were considered significant. Figure S16. Spatiotemporal dynamics of the PRRSV-2 1C sub-lineage in North America. (A–E) The heatmap panel on the top right displays number of transition between different regions, represented by color gradients and numerical values within each cell. Warm colors indicate high transition counts, while cool colors indicate low transitions counts. The thickness of the black boxes around the cells indicates the magnitude of the Bayes factor; only Bayes factors above 3 were considered significant. Figure S17. Spatiotemporal dynamics of the PRRSV-2 1E sub-lineage in North America. (A–E) The heatmap panel on the top right displays number of transition between different regions, represented by color gradients and numerical values within each cell. Warm colors indicate high transition counts, while cool colors indicate low transitions counts. The thickness of the black boxes around the cells indicates the magnitude of the Bayes factor; only Bayes factors above 3 were considered significant. Figure S18. Spatiotemporal dynamics of the PRRSV-2 1H sub-lineage in North America. (A–E) The heatmap panel on the top right displays number of transition between different regions, represented by color gradients and numerical values within each cell. Warm colors indicate high transition counts, while cool colors indicate low transitions counts. The thickness of the black boxes around the cells indicates the magnitude of the Bayes factor; only Bayes factors above 3 were considered significant. Table S1. Genetic distances measured as nucleotide identity percentages between newly identified Canadian lineages, undetermined Canadian clades, and all PRRSV-2 sub-lineages. Numbers outside the parentheses represent the medians for each comparison, and the numbers in parentheses represent the interquartile range. Nucleotide identity percentages were estimated using SDT v1.2. Table S2. Recombination events detected in the PRRSV-2. A. The analysis was performed encompassing the PRRSV-2 ORF-5. B. Only recombination events detected by at least four different methods and with P-values lower than a Bonferroni-corrected α = 0.05 were considered significant. Sub-lineages are indicated in parentheses for both the major and minor parental sequences. Table S3. Summary: This summary includes the count of each sub-lineage found in the respective Canadian provinces, the number of sequences related to vaccine-associated sub-lineages (when applicable), and the countries where these sub-lineages have been previously documented (based on GenBank sequence data). Country Abbreviations (ISO 2-letter codes): AT = Austria, CA = Canada, CO = Colombia, CL = Chile, CN = China, DE = Germany, DK = Denmark, ES = Spain, HK = Hong Kong, HU = Hungary, JP = Japan, KR = South Korea, LT = Lithuania, MX = Mexico, MM = Myanmar, MY = Malaysia, PE = Peru, PL = Poland, SG = Singapore, TH = Thailand, TW = Taiwan, US = United States. Vaccine-like N/A: Not applicable. Table S4. Distribution of intra- and inter-variant genetic distances of PRRSV-2. On the left, genetic distances within each variant are shown, represented by the first quartile (Q1), median, and third quartile (Q3). On the right, genetic distances between each variant and its closest related variant are presented, based on Q1 and median values. Table S5. Root-to-tip regression analysis performed in TempEst for each sub-lineage dataset. All five subsets were evaluated to assess evidence of temporal signal. We estimated the slope of the regression (interpreted as an approximate nucleotide substitution rate), the correlation coefficient between root-to-tip genetic distance and sampling time, and the coefficient of determination (R^2^). Table S6. Marginal likelihood estimates calculated for strict and relaxed molecular clock models for each dataset corresponding to the analyzed sub-lineages. Analyses were performed for each of the five subsampled datasets when applicable. Log marginal likelihoods were estimated using Generalized Stepping-Stone (GSS) sampling. Log Bayes factors (log BF) were calculated as the difference between the marginal likelihood estimates obtained for each molecular clock model. Table S7. Percentage nucleotide identity estimated within each dataset, including both the full dataset and each of the corresponding subsampled datasets (R1–R5) for each sub-lineage. Pairwise sequence comparisons were performed using SDT v1.2. Summary statistics include the mean and median percentage nucleotide identity (pident), first quartile (Q1), third quartile (Q3), and the minimum and maximum observed identity values.
