# Supplementary material for: Phylogenetic Lineages of PRRSV-2 from Canada Reveal Patterns of Transboundary Spread and Two Novel Sub-Lineages in North America
Source: Pathogens. 2026 Mar 24;15(4):346. doi: 10.3390/pathogens15040346 (PMC13118526; doi:10.3390/pathogens15040346)
Supplement: Supplementary file 1 [file pathogens-15-00346-s001.zip › pathogens-4114170-supplementary-final.pdf]

Supplement

Table S1. Genetic distances measured as nucleotide identity percentages between newly identified Canadian lineages, undetermined Canadian clades, and all PRRSV-2 sub-lineages. Numbers outside the parentheses represent the medians for each comparison, and the numbers in parentheses represent the interquartile range. Nucleotide identity percentages were estimated using SDT v1.2.

**Discussion Conclusion Reference**	**Genetic distances within and between clades (median, IQR)**			
	**1L**	**1K**	**Undetermined 1**	**Undetermined 2**
**1L**	6.5 (4-9.4)			
**1K**	12.7 (11.7-13.7)	10.4 (7.6-12.1)		
**Undetermined 1**	10.3 (9.4-11.1)	10.8 (9.6-11.8)	5.3 (3.8-6.5)	
**Undetermined 2**	11.3 (10.4-12.5)	12.1 (11.1-13.1)	9.1 (8.3-9.9)	6.5 (3.6-8.1)
**1A**	13.1 (12.4-13.8)	13.3 (12.6-14.1)	11.4 (10.8-11.9)	12.3 (11.8-12.9)
**1B**	14.8 (14.1-15.4)	14.6 (13.8-15.2)	12.9 (12.4-13.6)	13.6 (12.8-14.3)
**1C**	12.3 (11.4-13.1)	13.1 (12.1-13.8)	10.6 (9.9-11.3)	11.9 (11.1-12.8)
**1D**	11.8 (11.1-12.3)	13 (12.3-13.9)	10.9 (10.6-11.4)	12.1 (11.6-12.7)
**1E**	14.1 (13.2-15.1)	14.7 (13.7-15.5)	13.6 (12.5-14.3)	14.4 (13.4-15.2)
**1F**	11.1 (10.3-11.9)	11.6 (10.4-12.6)	9.5 (8.7-10.3)	10.9 (10.1-11.9)
**1H**	13.4 (12.6-14.3)	14.2 (13.2-15.2)	13.2 (12.6-13.9)	14.4 (13.7-15.1)
**1I**	13.9 (13.4-14.4)	14.2 (13.3-14.9)	12.4 (11.9-12.9)	12.6 (12.1-13.4)
**1J**	14.1 (13.3-14.8)	14.2 (13.1-15.1)	12.3 (11.6-13.1)	13.7 (13.1-14.4)
**2**	14.7 (13.9-15.8)	15.7 (14.7-16.5)	14.6 (14-15.2)	16 (15.2-16.6)
**3**	16.2 (15.4-16.9)	16.5 (15.3-17.7)	15.6 (14.7-16.6)	16.1 (15.2-17.2)
**4**	13.6 (12.5-14.5)	13.7 (12.6-14.8)	12.4 (11.4-13.3)	13.9 (13.1-14.7)
**5A**	15.4 (14.7-16)	15 (14.5-15.7)	13.7 (13.2-14.2)	15.3 (14.9-15.7)
**5B**	14.9 (14.4-15.6)	16 (15.2-17.2)	14.6 (14.1-15)	16.1 (15.6-17)
**6**	16.3 (15.5-17.3)	16.3 (15.5-16.9)	14.5 (13.9-15.1)	16.8 (16.2-17.9)

**7**	14.7 (14.1-15.2)	13.9 (13.2-14.6)	12.7 (12.3-13.2)	14.9 (13.9-15.3)
**8A**	14.6 (13.7-15.2)	15.5 (14.8-16.3)	14.2 (13.9-14.7)	15.4 (14.9-16.4)
**8B**	15.3 (14.5-16)	15 (14.2-16)	14.4 (13.8-15)	15.3 (14.8-16)
**8C**	14.7 (14-15.5)	14.7 (13.9-15.5)	13.4 (12.9-13.9)	15.5 (15.2-16)
**8D**	17.5 (17-18)	17.5 (16.9-18.3)	16.5 (16.2-16.9)	18.5 (18-18.9)
**8E**	14.7 (13.7-15.5)	14.7 (14.1-15.5)	13.7 (13-14.3)	15.2 (14.5-15.7)
**9A**	15.4 (14.6-16.1)	15.2 (14.4-15.9)	14.4 (13.6-15.1)	15.8 (15.1-16.5)
**9B**	15 (14.4-15.5)	14.9 (14.2-15.7)	14.1 (13.4-14.9)	15.8 (15.2-16.4)
**9C**	15.4 (14.7-16.1)	15.4 (14.8-16.2)	15.1 (14.4-15.7)	15.9 (15.1-16.6)
**9D**	15.9 (15.2-16.8)	15.4 (14.6-16.1)	15.3 (14.6-15.9)	15.8 (15.1-16.8)
**10**	14.9 (14-15.8)	14.9 (14.4-15.6)	14.4 (13.6-14.9)	15.5 (15.2-16)
**11**	14.7 (13.9-15.5)	15.3 (14.4-16.1)	14 (13.3-14.8)	15 (14.2-15.7)

Table S2. Recombination events detected in the PRRSV-2. A. The analysis was performed encompassing the PRRSV-2 ORF-5. B. Only recombination events detected by at least four different methods and with P-values lower than a Bonferroni-corrected α = 0.05 were considered significant. Sub-lineages are indicated in parentheses for both the major and minor parental sequences.

# Abbreviations of detection methods R, Rdp; G, Geneconv; B, Boostcan; M, Maxichi; C, Chimaera; S, Siscan; 3, 3Seq.

* Lowest P-value reported by the method in bold and underline

		Breakpoint Position In Recombinant sequence						
Event	Found in	Begin	End	Recombination sequences	Minor Parental	Major Parental	Detection Methods #	P-value*
1	6	358	435	5734 (1L)	Unknown	5254 (1L)	MC**S**T	$5.221e^{-05}$
2	21	355	597	5518 (1L)	6249 1L	288 (1L)	MC**S**T	$4.325e^{-03}$

1	1	32	96	2019 (1K)	Unknown	1101 (1K)	RGBMC**T**	1.759e^{-09}

Table S3. Summary: This summary includes the count of each sub-lineage found in the respective Canadian provinces, the number of sequences related to vaccine-associated sub-lineages (when applicable), and the countries where these sub-lineages have been previously documented (based on GenBank sequence data). Country Abbreviations (ISO 2-letter codes): AT = Austria, CA = Canada, CO = Colombia, CL = Chile, CN = China, DE = Germany, DK = Denmark, ES = Spain, HK = Hong Kong, HU = Hungary, JP = Japan, KR = South Korea, LT = Lithuania, MX = Mexico, MM = Myanmar, MY = Malaysia, PE = Peru, PL = Poland, SG = Singapore, TH = Thailand, TW = Taiwan, US = United States. Vaccine-like N/A: Not applicable

Sub-lineage	Total	Quebec	Ontario	Manitoba	Alberta	Saskatchewan	Vaccine-like	Other countries
1A	6	-	-	-	6	-	N/A	CA, CN, CO, KR, MX, PE, TW, US
1B	53	16	37	-	-	-	N/A	CA, CN, KR, CL, MX, US
1C	133	133	-	-	-	-	N/A	CA, CN, KR, US
1E	97	89	8	-	-	-	N/A	CA, MX, HU, KR, US,
1F	9	6	3	-	-	-	-	CA, US, JP
1H	1064	908	156	-	-	-	N/A	CA, MX, US
1I	33	28	5		-	-	N/A	CA, TH, US
1K	239	141	97	1	-	-	N/A	CA, US

1L	303	303	-	-	-	-	N/A	CA
5A	1078	664	133	271	5	5	959	AT, CA, CN, DE, DK, ES, HK, HU, KR, LT, MX, MY, PL, SG, TH, TW, US, VN
7	10	10	-	-	-	-	10	CA, CN, US
8A	83	53	4	26	-	-	83	CA, MX, US
8C	50	34	-	16	-	-	50	CA, CN, MX, US
9A	1	1	-	-	-	-	1	CA, MX, US
Und-1	55	55	-	-	-	-	N/A	CA
Und-2	49	49	-	-	-	-	N/A	CA

Table S4. Distribution of intra- and inter-variant genetic distances of PRRSV-2. On the left, genetic distances within each variant are shown, represented by the first quartile (Q1), median, and third quartile (Q3). On the right, genetic distances between each variant and its closest related variant are presented, based on Q1 and median values.

Genetic distance within variant				Genetic distance in relation to the closet related variant			
Variant	Q1(25th percentile)	Median	Q3 (25th percentile)	Variant	Q1(25th percentile)	Median	Q3 (25th percentile)
1C.41	4.64	7.16	8.50	1C.42	9.28	10.00	10.94
1C.42	1.82	3.31	4.14	1C.41	9.28	10.00	10.94
1E.10	3.10	4.72	6.96	1E.4	7.79	8.66	9.61
1E.11	1.49	2.65	3.81	1E.4	9.28	9.87	10.56
1E.12	1.99	3.15	4.80	1E.4	5.43	6.72	7.94
1K.1	4.64	6.16	7.13	1K.3	6.95	7.85	8.78
1K.2	6.30	8.29	10.42	1K.3	9.95	10.91	11.77
1K.4	3.81	7.29	7.46	1K.3	8.29	9.01	9.45
1L.1	2.48	5.47	6.79	1L.2	6.96	7.96	8.78
1L.2	1.99	2.98	3.81	1L.4	6.63	7.13	7.62
1L.3	0.66	1.49	2.48	1L.4	6.13	6.30	6.63
1L.4	0.20	0.33	0.49	1L.3	6.13	6.30	6.63
1H.36	2.65	3.81	5.14	1H.38	5.97	6.46	7.13
1H.37	3.10	4.64	5.80	1H.39	5.80	6.79	7.62
1H.38	1.69	2.65	3.81	1H.46	4.97	5.63	5.97
1H.39	2.15	3.31	4.64	1H.41	5.30	5.97	6.96
1H.40	1.40	2.90	3.35	1H.45	5.80	6.63	7.13
1H.41	1.11	2.32	3.31	1H.39	5.30	5.97	6.96
1H.42	2.81	4.64	5.63	1H.39	5.63	6.63	7.79
1H.43	2.81	5.80	7.29	1H.41	7.96	8.62	9.28
1H.44	1.16	2.15	3.31	1H.38	7.13	7.96	8.62
1H.45	1.82	3.48	4.47	1H.49	5.80	6.46	7.29
1H.46	1.99	2.98	4.47	1H.38	4.97	5.63	5.97
1H.47	1.65	2.48	3.31	1H.42	7.62	8.29	9.28
1H.48	2.15	3.31	4.47	1H.49	7.62	8.12	8.62
1H.49	1.82	2.65	4.14	1H.36	6.13	6.96	7.79

Table S5. Root-to-tip regression analysis performed in TempEst for each sub-lineage dataset. All five subsets were evaluated to assess evidence of temporal signal. We estimated the slope of the regression (interpreted as an approximate nucleotide substitution rate), the correlation coefficient between root-to-tip genetic distance and sampling time, and the coefficient of determination (R^2).

Sub-lin	slope (rates)	correlation coefficient	r^2
1A_R1	4.03E-03	0.60	0.36
1A_R2	4.39E-03	0.64	0.41
1A_R3	4.15E-03	0.63	0.4
1A_R4	4.13E-03	0.63	0.39
1A_R5	3.80E-03	0.61	0.37
1B_R1	5.88-E03	0.52	0.26
1B_R2	5.59E-03	0.71	0.49
1B_R3	4.34E-03	0.70	0.49
1B_R4	4.56E-03	0.72	0.52
1B_R5	4.40E-03	0.71	0.5
1C_R1	7.96E-03	0.69	0.47
1C_R2	5.10E-03	0.73	5.36
1C_R3	5.01E-03	0.74	0.55
1C_R4	4.42E-03	0.69	0.48
1C_R5	5.17E-03	0.80	0.63
1E_R1	5.98E-03	0.84	0.71
1E_R2	5.99E-03	0.84	0.71
1E_R3	5.97E-03	0.84	0.71
1E_R4	5.97E-03	0.84	0.71
1E_R5	5.98E-00	0.84	0.71
1F	3.58E-03	0.46	0.21
1H_R1	7.43E-03	0.71	0.51
1H_R2	5.01E-03	0.61	0.38
1H_R3	5.22E-03	0.62	0.39
1H_R4	5.08E-03	0.61	3.69
1H_R5	5.17E-03	0.62	0.39
1I	6.15E-03	0.66	0.43
1K	4.45E-03	0.77	0.58

Table S6. Marginal likelihood estimates calculated for strict and relaxed molecular clock models for each dataset corresponding to the analyzed sub-lineages. Analyses were performed for each of the five subsampled datasets when applicable. Log marginal likelihoods were estimated using Generalized Stepping-Stone (GSS) sampling. Log Bayes factors (log BF) were calculated as the difference between the marginal likelihood estimates obtained for each molecular clock model.

Sub-lin	Strict Clock	Relaxed clock	Log Bayes factor
1A_R1	-32400.72	-32368.89	31.83
1A_R2	-32321.28	-32247.39	73.89
1A_R3	-32616.95	-32550.2	66.75
1A_R4	-32418.22	-32363.39	54.83
1A_R5	-32224.3	-32161.06	63.24
1B_R1	-24708.14	-24700.42	7.72
1B_R2	-20218.83	-20122.56	96.27
1B_R3	-23160.72	-23041.34	119.38
1B_R4	-22534.26	-22503.13	31.13
1B_R5	-20176.57	-20168.69	7.88
1C_R1	-36079.07	-36061.53	17.54
1C_R2	-33527.8	-33498.74	29.06
1C_R3	-33335.27	-33269.6	65.67
1C_R4	-33203.77	-33202.04	1.73
1C_R5	-33392.35	-33355.5	36.85
1E_R1	-18551.12	-18523.4	27.72
1E_R2	-15851.25	-15805.34	45.91
1E_R3	-15840.93	-15814.11	26.82
1E_R4	-15842.64	-15795.08	47.56
1E_R5	-15823.36	-15802.35	21.01
1F	-6826.79	-6781.02	45.77
1H_R1	-35103.99	-34990.65	113.34
1H_R2	-35778.78	-35683.24	95.54
1H_R3	-36689.39	-36628.62	60.77
1H_R4	-36685.91	-36604.38	81.53
1H_R5	-35819.45	-35759.15	60.3

1I	-3141.54	-3135.39	6.15
1K	-12861.71	-12836.81	24.9

Table S7. Percentage nucleotide identity estimated within each dataset, including both the full dataset and each of the corresponding subsampled datasets (R1–R5) for each sub-lineage. Pairwise sequence comparisons were performed using SDT v1.2. Summary statistics include the mean and median percentage nucleotide identity (pident), first quartile (Q1), third quartile (Q3), and the minimum and maximum observed identity values.

Data_set	mean_pident	median_pident	q1_pident	q3_pident	min_pident	max_pident
1A_Full_data	96.36	96.51	95.35	97.51	87.56	100
1A_R1	94.53	94.53	93.7	95.52	87.58	99.83
1A_R2	94.55	94.54	93.7	95.52	87.58	99.83
1A_R3	94.56	94.54	93.7	95.52	87.58	99.83
1A_R4	94.54	94.53	93.7	95.52	87.58	99.83
1A_R5	94.55	94.54	93.7	95.52	87.58	99.83
1B_Full_data	94.49	94.37	92.86	96.18	85.90	100
1B_R1	93.12	93.04	91.71	94.53	86.07	100
1B_R2	93.85	93.71	92.39	95.36	86.26	100
1B_R3	93.86	93.71	92.4	95.36	86.26	100
1B_R4	93.85	93.71	92.39	95.36	86.26	100
1B_R5	93.84	93.71	92.38	95.36	86.26	100
1C_Full_data	93.13	92.88	91.15	94.85	83.25	100
1C_R1	90.62	90.41	88.89	92.37	82.62	100
1C_R2	91.39	91.21	89.55	93.37	82.62	100

1C_R3	91.36	91.06	89.48	93.21	82.62	100
1C_R4	91.36	91.06	89.42	93.21	82.62	100
1C_R5	91.37	91.06	89.55	93.27	82.62	100
1E_Full_data	92.58	93.69	89.90	96.02	80.03	100
1E_R1	91.15	91.71	87.75	94.31	80.03	100
1E_R2	91.95	92.04	89.72	94.69	82.09	100
1E_R3	91.95	92.04	89.72	94.69	82.09	100
1E_R4	91.95	92.04	89.72	94.69	82.09	100
1E_R5	91.95	92.04	89.72	94.69	82.09	100
1F	93.48	93.2	92.04	94.53	86.24	100
1H_Full_data	92.73	92.70	91.06	94.19	82.11	100
1H_R1	91.38	91.38	90.05	92.7	84.27	100
1H_R2	91.37	91.23	90.05	92.55	84.74	100
1H_R3	91.4	91.36	90.05	92.57	84.74	100
1H_R4	91.42	91.38	90.07	92.7	84.74	100
1H_R5	91.37	91.23	90.05	92.55	84.74	100
1I	92.42	91.05	88.41	96.52	86.42	100
1K	90.46	89.88	88.23	92.7	82.92	100

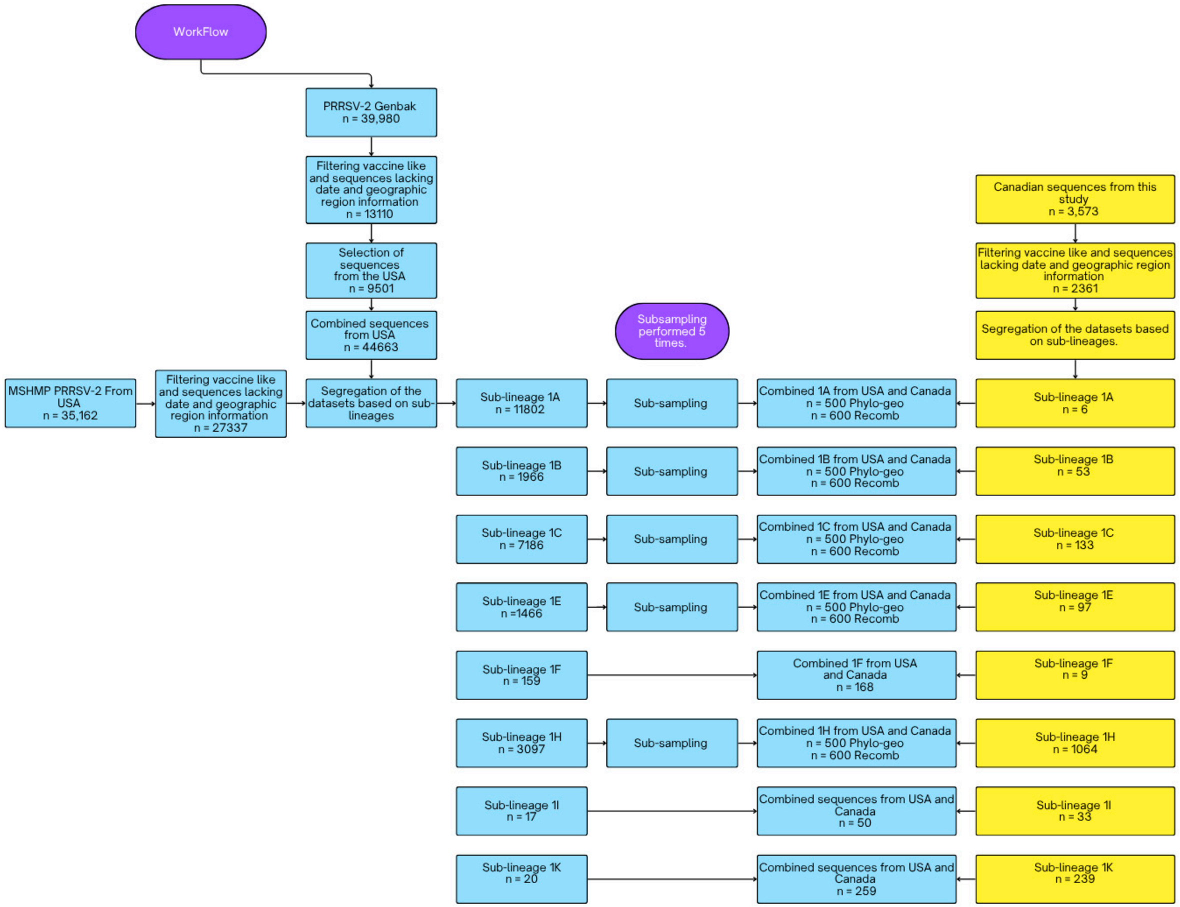

Figure S1. Workflow of PRRSV-2 dataset processing and sub-lineage segregation. Sequences were retrieved from GenBank (n = 39,980), MSHMP (n = 35,162), and Canadian sequences generated in this study (n = 3,573). After filtering GenBank sequences lacking collection date or geographic region (n = 13,110), U.S. sequences (n = 9,501) and Canadian sequences (n = 118) were selected. Combined datasets from the USA and Canada were subsequently segregated by sub-lineage. For each major sub-lineage (1A, 1B, 1C,

1E, 1F, 1H, 1I, and 1K), subsampling was performed five times to generate representative datasets for phylogeographic (n = 500) and recombination (n = 600) analyses. The left and right panel shows the distribution of Canadian sequences across sub-lineages after segregation and subsampling.

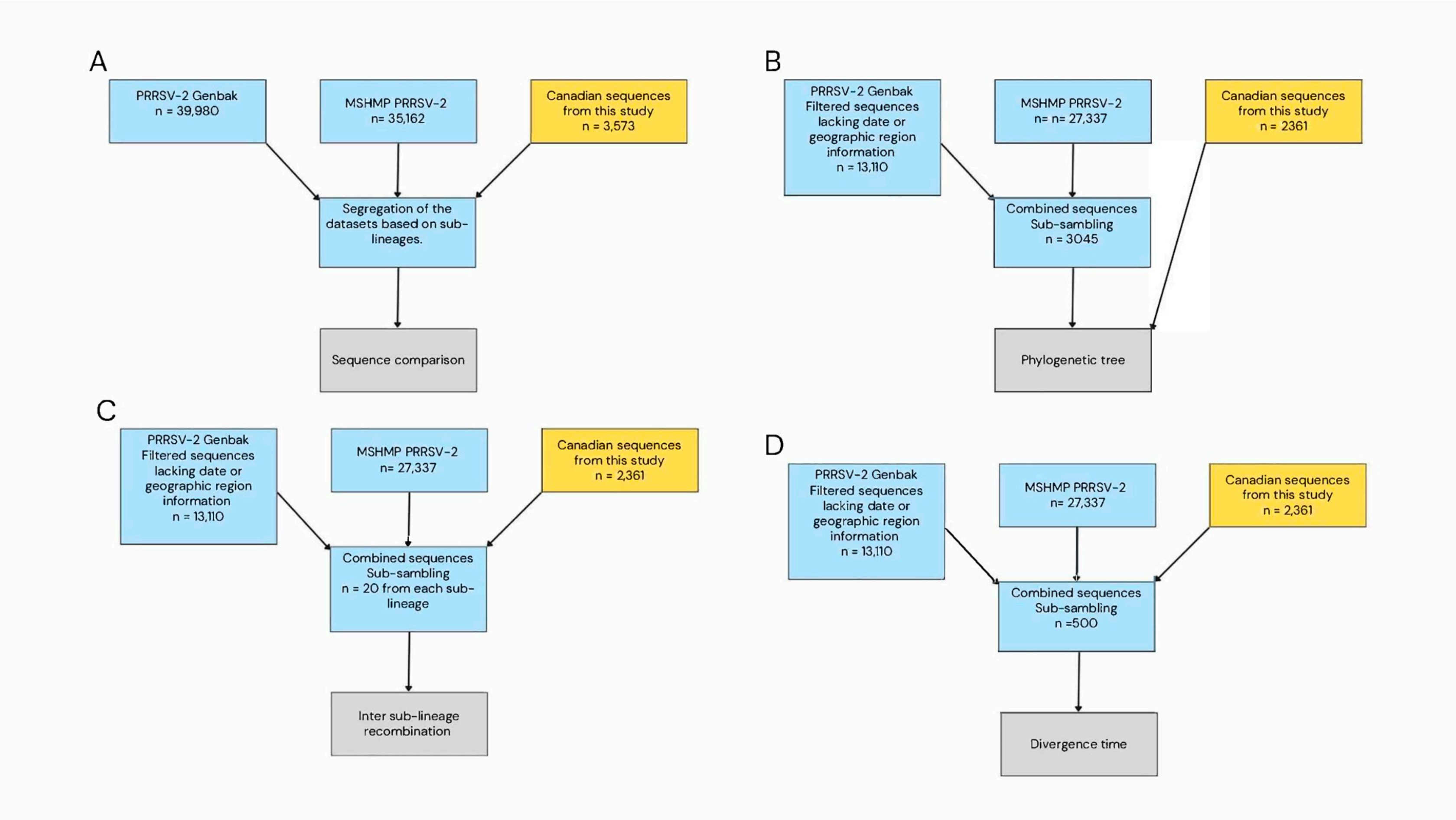

Figure S2. Workflow of sequence datasets and analyses. (A) Segregation of PRRSV-2 sequences from GenBank (n = 39,980), MSHMP (n = 35,162), and Canadian sequences from this study (n = 3,354) based on sub-lineages for sequence comparison. (B) Construction of a phylogenetic tree using a combined dataset after filtering GenBank sequences lacking date or geographic region information (n = 13,110) and sub-sampling (n = 3,045). (C) Assessment of inter–sub-lineage recombination using a combined dataset with sub-sampling (n = 20 per sub-lineage). (D) Estimation of divergence times using a combined dataset with sub-sampling (n = 500).

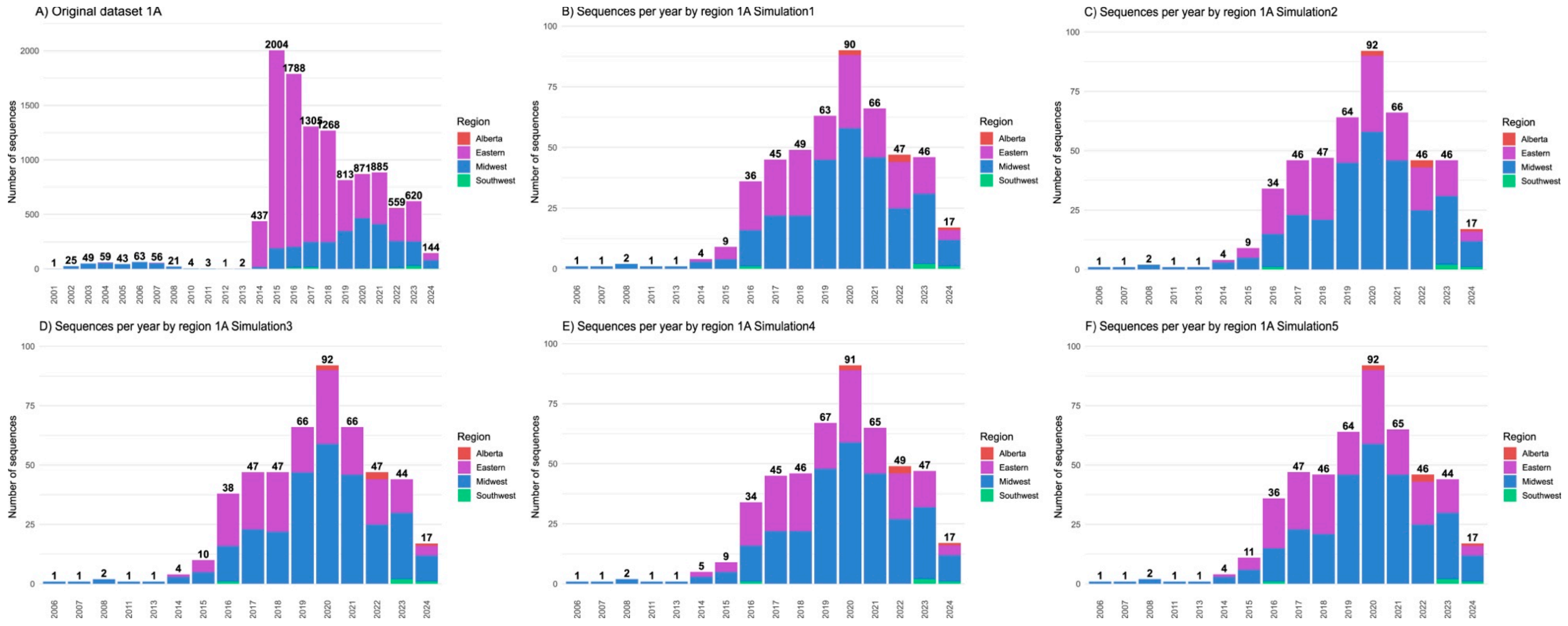

Figure S3.(A) Original dataset of sub-lineage 1A sequences. (B–F) Sub-sampled datasets from five independent simulations showing the number of sequences per year across regions in the USA and Canada. Sub-sampling was performed to standardize sample size across years, geographic regions, and genetic distances.

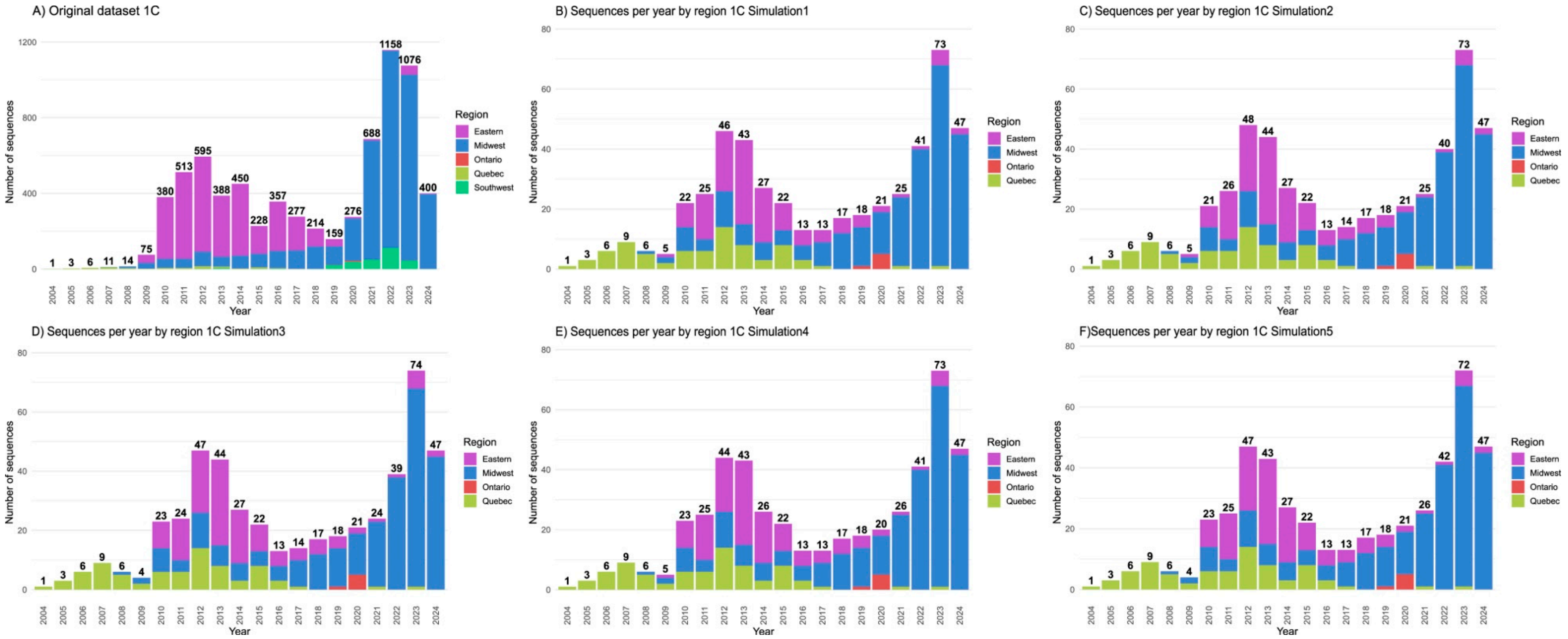

Figure S4.(A) Original dataset of sub-lineage 1C sequences. (B–F) Sub-sampled datasets from five independent simulations showing the number of sequences per year across regions in the USA and Canada. Sub-sampling was performed to standardize sample size across years, geographic regions, and genetic distances.

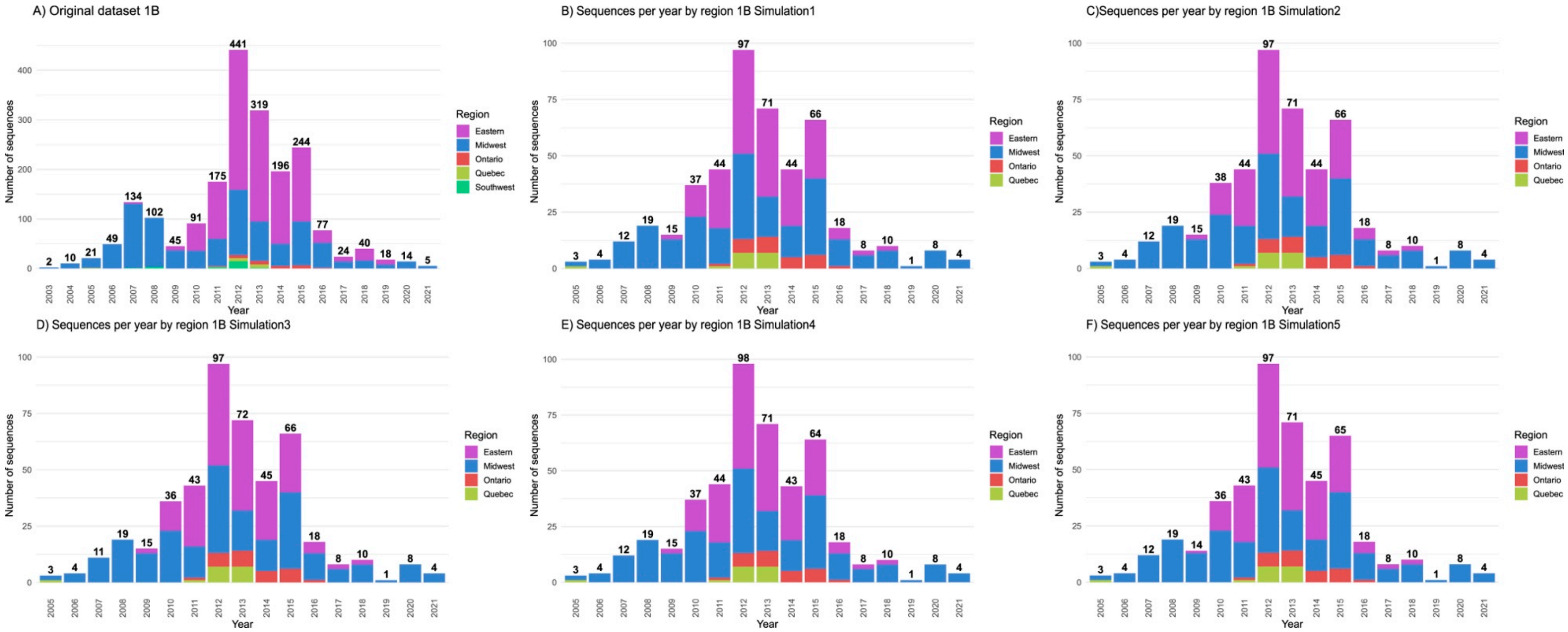

Figure S5.(A) Original dataset of sub-lineage 1B sequences. (B–F) Sub-sampled datasets from five independent simulations showing the number of sequences per year across regions in the USA and Canada. Sub-sampling was performed to standardize sample size across years, geographic regions, and genetic distances.

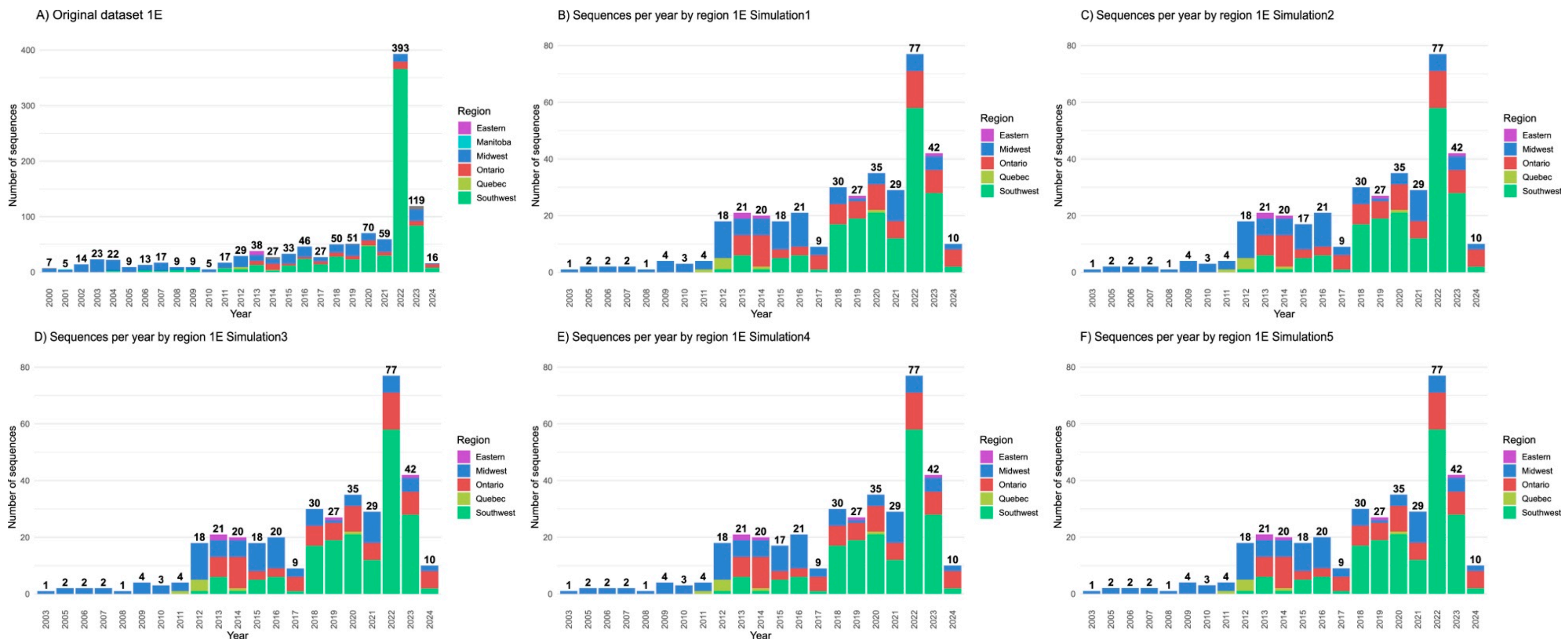

Figure S6.(A) Original dataset of sub-lineage 1E sequences. (B–F) Sub-sampled datasets from five independent simulations showing the number of sequences per year across regions in the USA and Canada. Sub-sampling was performed to standardize sample size across years, geographic regions, and genetic distances.

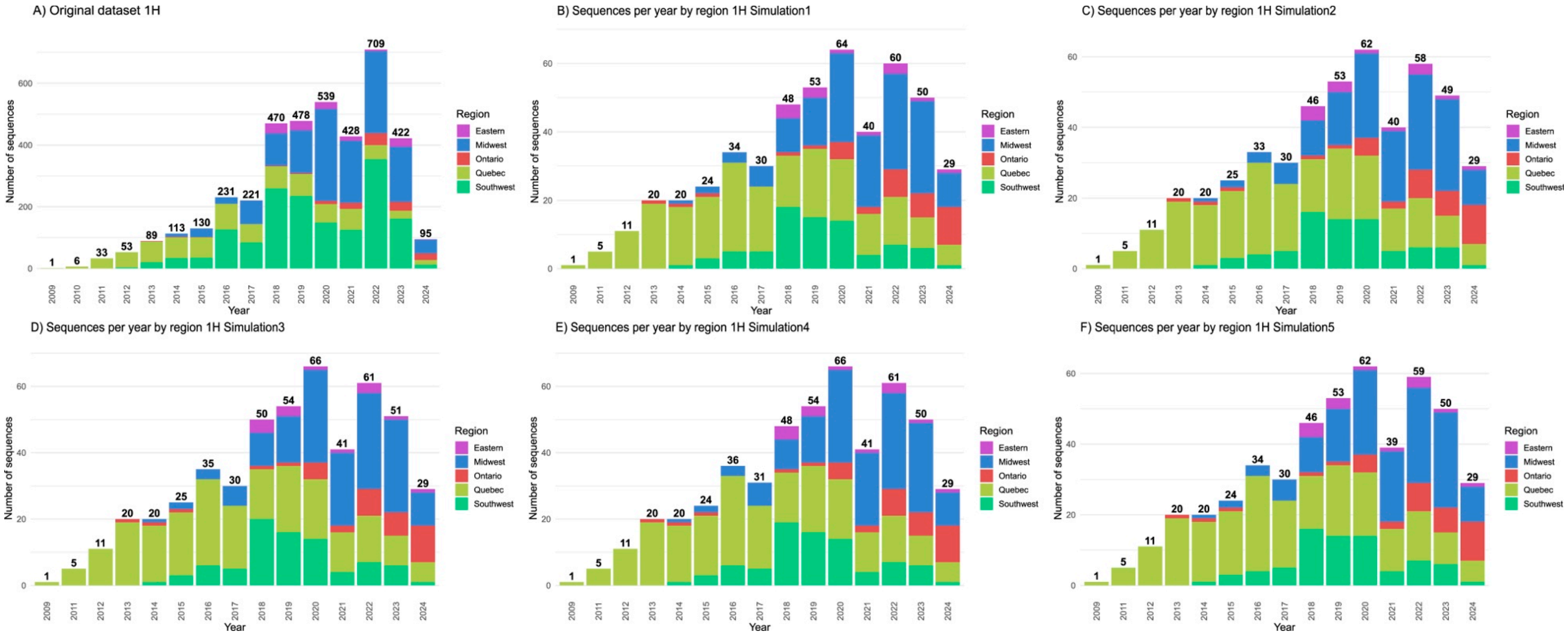

Figure S7.(A) Original dataset of sub-lineage 1H sequences. (B–F) Sub-sampled datasets from five independent simulations showing the number of sequences per year across regions in the USA and Canada. Sub-sampling was performed to standardize sample size across years, geographic regions, and genetic distances.

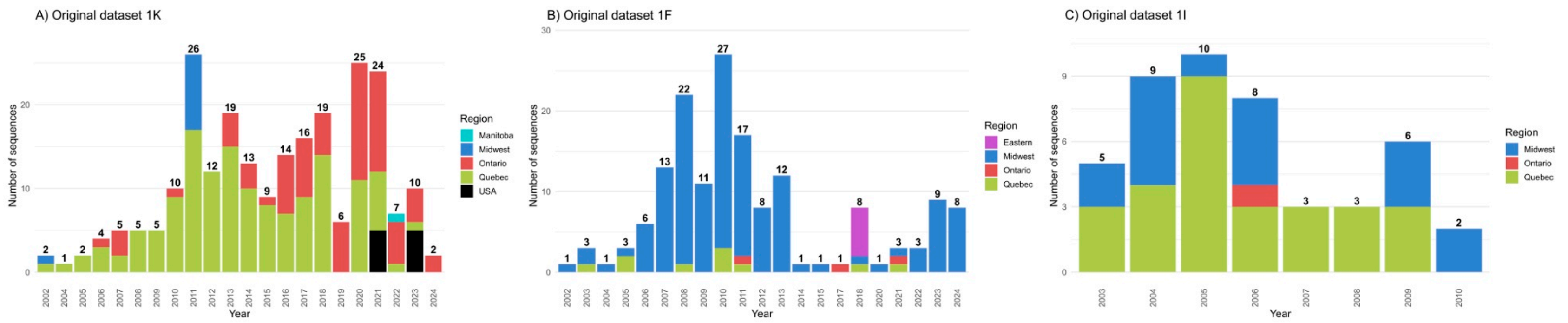

Figure S8. Temporal distribution of PRRSV-2 sequences in Canada for three original datasets. (A) Sub-lineage 1K, (B) sub-lineage 1F, and (C) sub-lineage 1I. Bars represent the number of sequences collected per year, stratified by geographic region (Eastern, Western, Ontario, Quebec, and Atlantic). Numbers above the bars indicate the count of sequences for each year–region combination

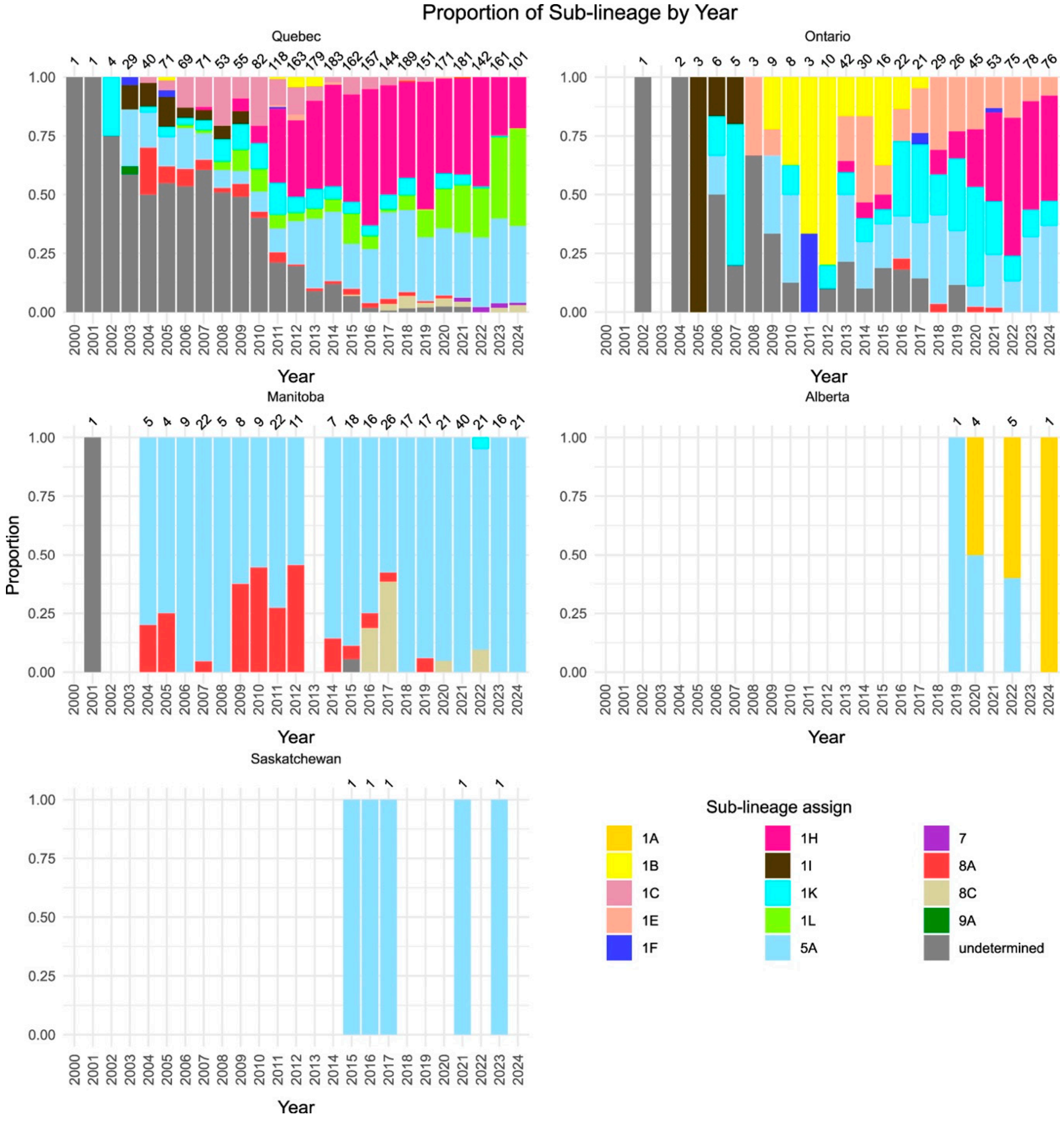

Figure S9. Proportion of PRRSV-2 ORF5 sequences per year by province. Each panel represents a Canadian province. The bars show the proportion of each sub-lineage over time within each province, with the number on top of each bar indicating the total number of sequences represented by that bar. The different colors represent the various sub-lineages.

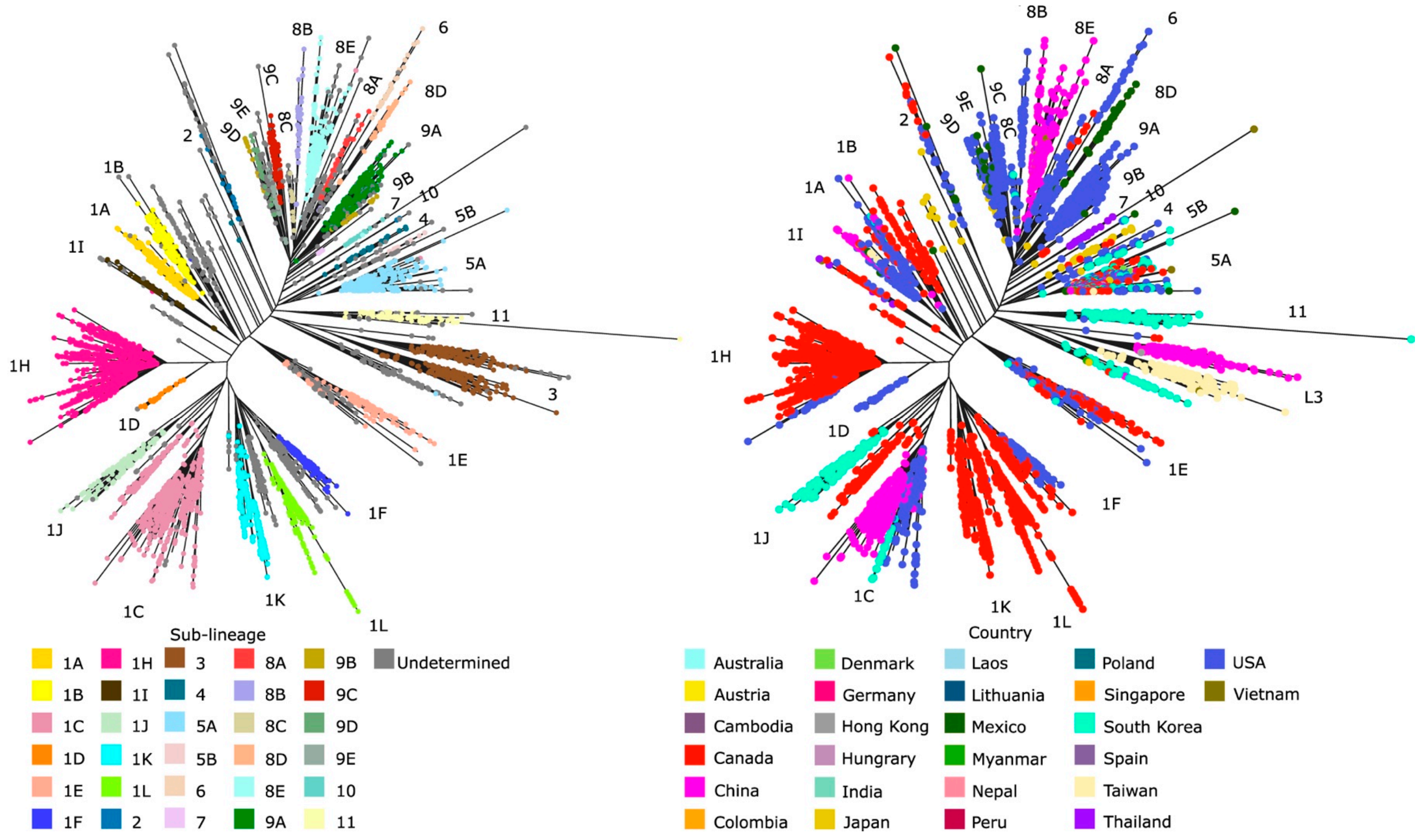

Figure S10. Unrooted phylogenetic trees reconstructed using maximum likelihood. The phylogenetic trees were generated using PRRSV-2 ORF-5 sequences from a dataset comprising over 6,000 global sequences, representing the diversity within each PRRSV-2 sub-lineage. Both panels display the same phylogenetic tree. A) Each color represents a different PRRSV-2 sub-lineage. B) Each color represents the geographic origin of each sequence. The phylogenies were reconstructed using RAxML-NG with the GTR+I+G4 nucleotide substitution model.

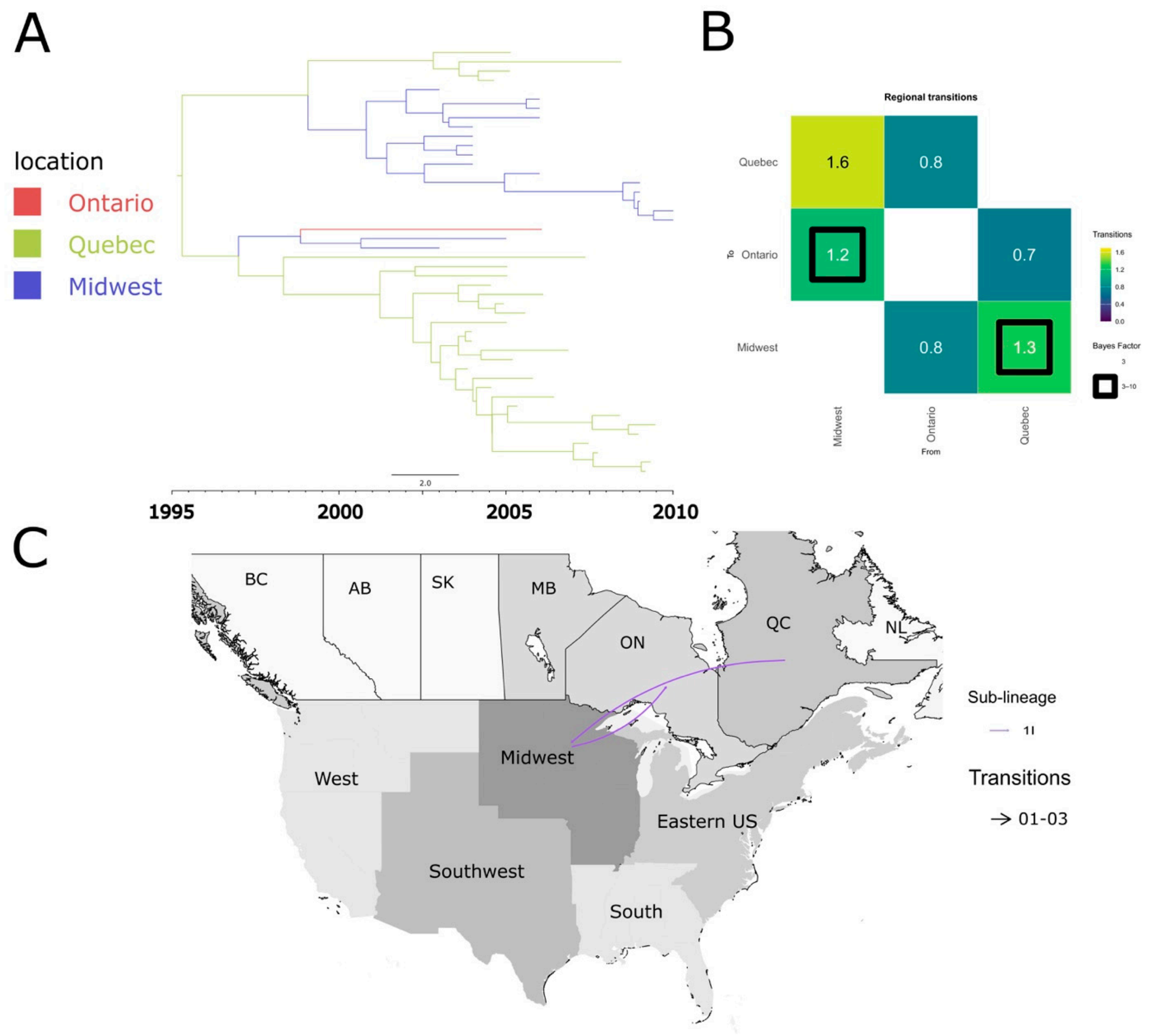

Figure S11. Spatiotemporal dynamics of the PRRSV-2 1I sub-lineage in North America. A) The panel on the top left shows a maximum clade credibility (MCC) phylogenetic tree reconstructed using a discrete-space diffusion model. B) The heatmap panel on the top right displays number of transition between different regions, represented by color gradients and numerical values within each cell. Warm colors indicate high transition counts, while cool colors indicate low transitions counts. The thickness of the black boxes around the cells indicates the magnitude of the Bayes factor; only Bayes factors above 3 were considered significant. C) The bottom panel shows spread pathway. Arrows indicate the direction of spread, and their thickness represents the transition numbers. The USA regions shown correspond to swine-producing areas, defined based on animal density according to SHIC. The abbreviations on the Canada map refer to different provinces.

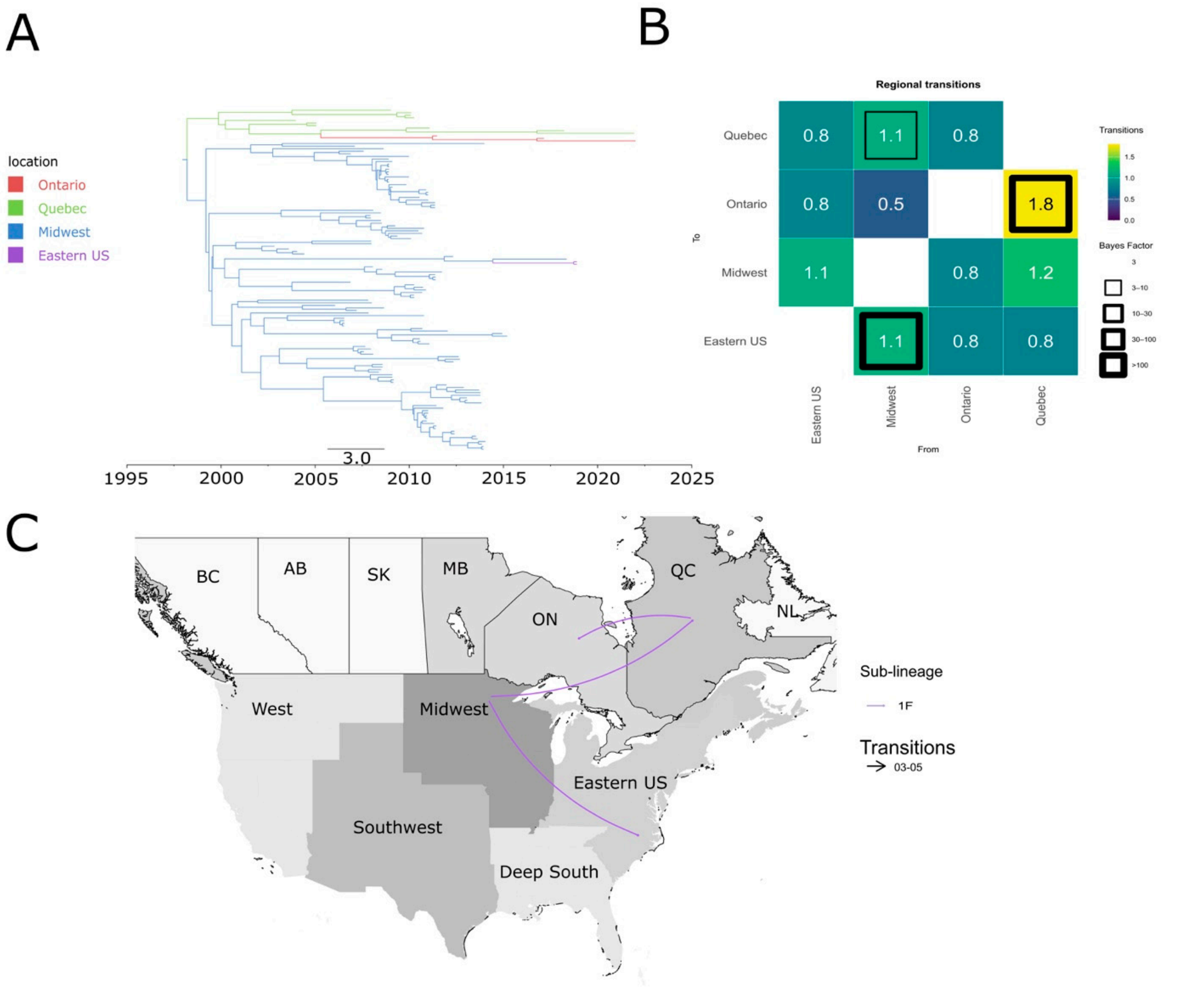

Figure S12. Spatiotemporal dynamics of the PRRSV-2 1F sub-lineage in North America. A) The panel on the top left shows a maximum clade credibility (MCC) phylogenetic tree reconstructed using a discrete-space diffusion model. B) The heatmap panel on the top right displays number of transition between different regions, represented by color gradients and numerical values within each cell. Warm colors indicate high transition counts, while cool colors indicate low transitions counts. The thickness of the black boxes around the cells indicates the magnitude of the Bayes factor; only Bayes factors above 3 were considered significant. C) The bottom panel shows spread pathway. Arrows indicate the direction of spread, and their thickness represents the transition numbers. The USA regions shown correspond to swine-producing areas, defined based on animal density according to SHIC. The abbreviations on the Canada map refer to different provinces.

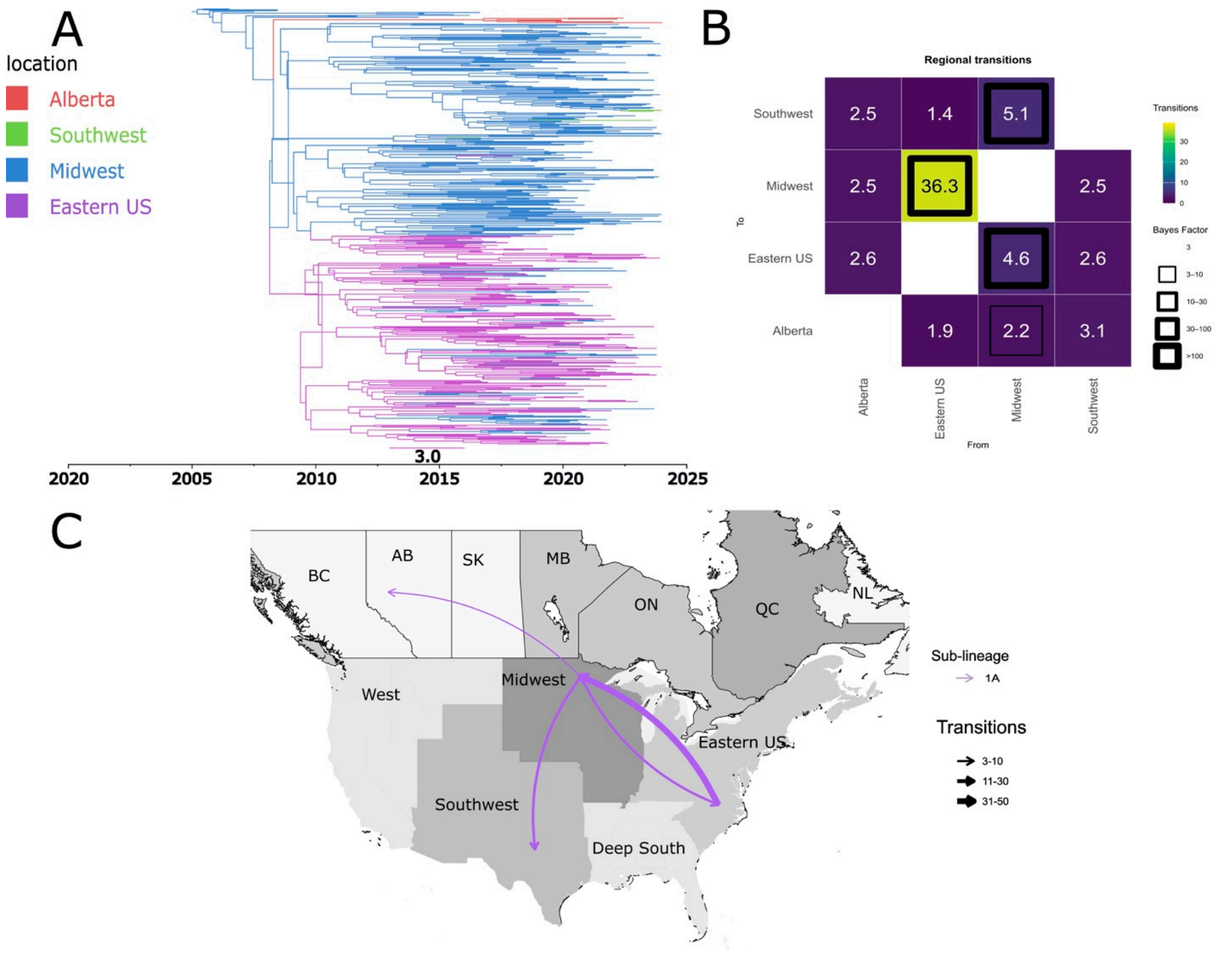

Figure S13. Spatiotemporal dynamics of the PRRSV-2 1A sub-lineage in North America. A) The panel on the top left shows a maximum clade credibility (MCC) phylogenetic tree reconstructed using a discrete-space diffusion model. B) The heatmap panel on the top right displays number of transition between different regions, represented by color gradients and numerical values within each cell. Warm colors indicate high transition counts, while cool colors indicate low transitions counts. The thickness of the black boxes around the cells indicates the magnitude of the Bayes factor; only Bayes factors above 3 were considered significant. C) The bottom panel shows spread pathway. Arrows indicate the direction of spread, and their thickness represents the transition numbers. The USA regions shown correspond to swine-producing areas, defined based on animal density according to SHIC. The abbreviations on the Canada map refer to different provinces.

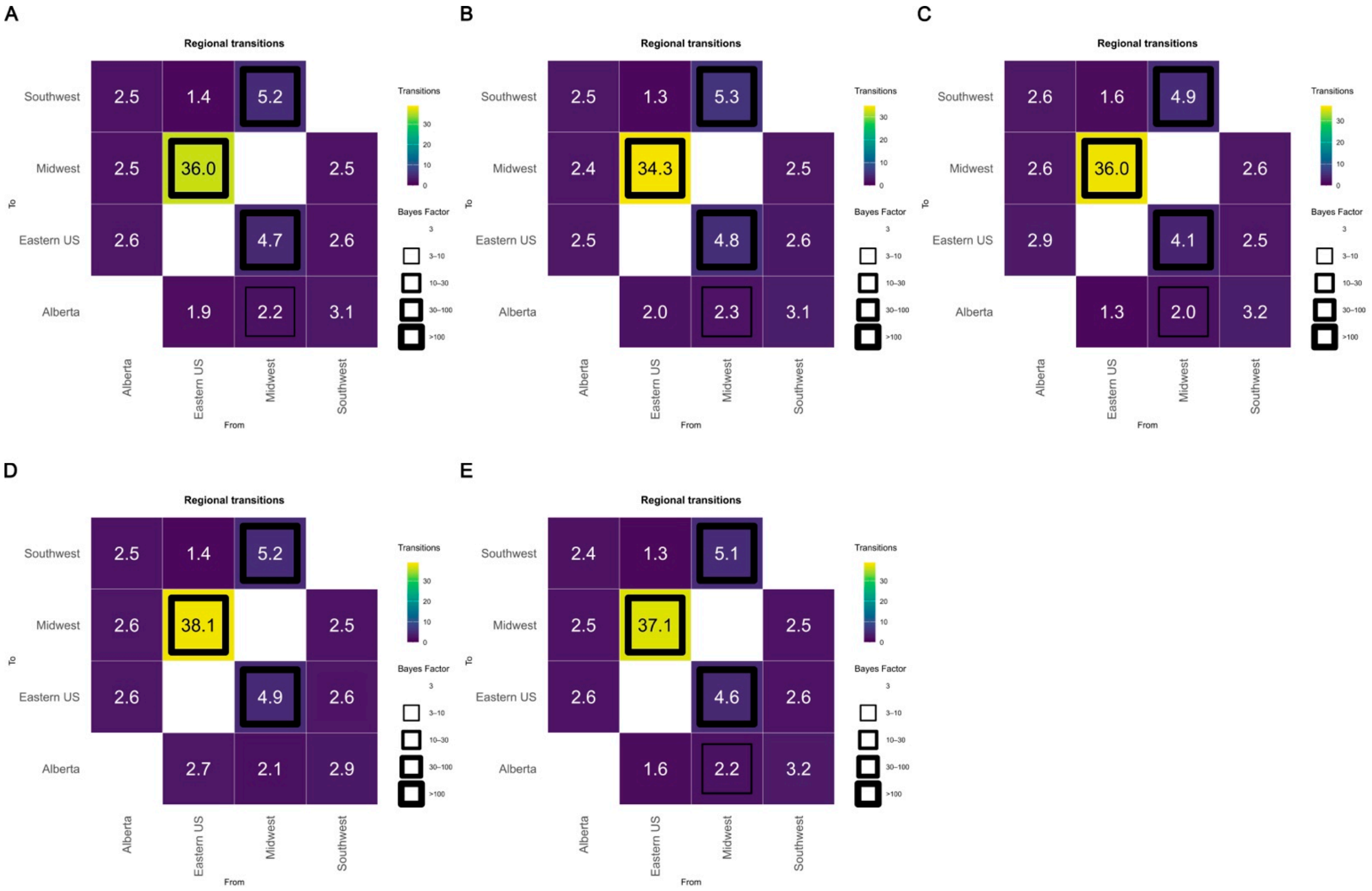

Figure S14. Spatiotemporal dynamics of the PRRSV-2 1A sub-lineage in North America. A to E) The heatmap panel on the top right displays number of transition between different regions, represented by color gradients and numerical values within each cell. Warm colors indicate high transition counts, while cool colors indicate low transitions counts. The thickness of the black boxes around the cells indicates the magnitude of the Bayes factor; only Bayes factors above 3 were considered significant.

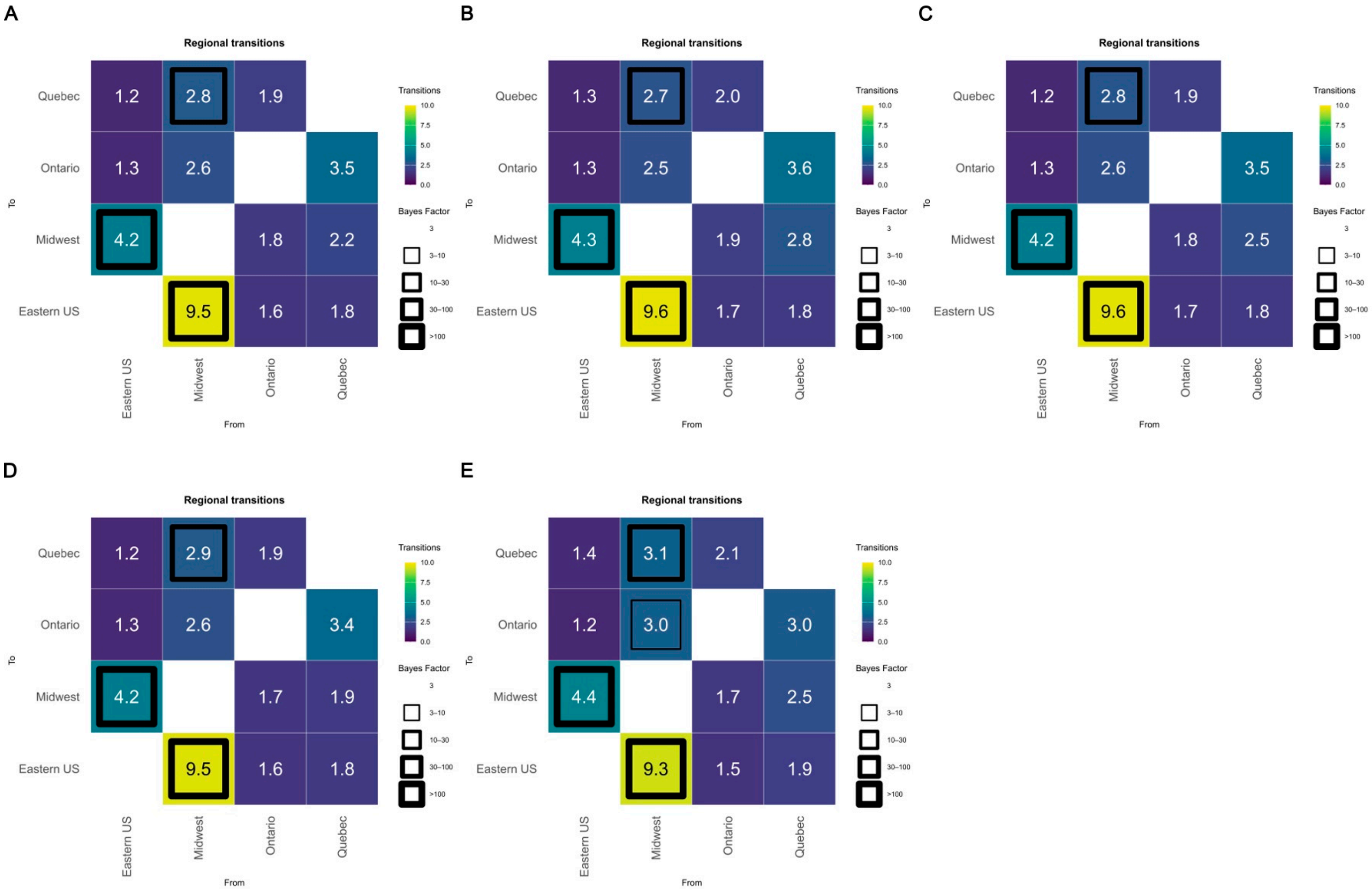

Figure S15. Spatiotemporal dynamics of the PRRSV-2 1B sub-lineage in North America. A to E) The heatmap panel on the top right displays number of transition between different regions, represented by color gradients and numerical values within each cell. Warm colors indicate high transition counts, while cool colors indicate low transitions counts. The thickness of the black boxes around the cells indicates the magnitude of the Bayes factor; only Bayes factors above 3 were considered significant.

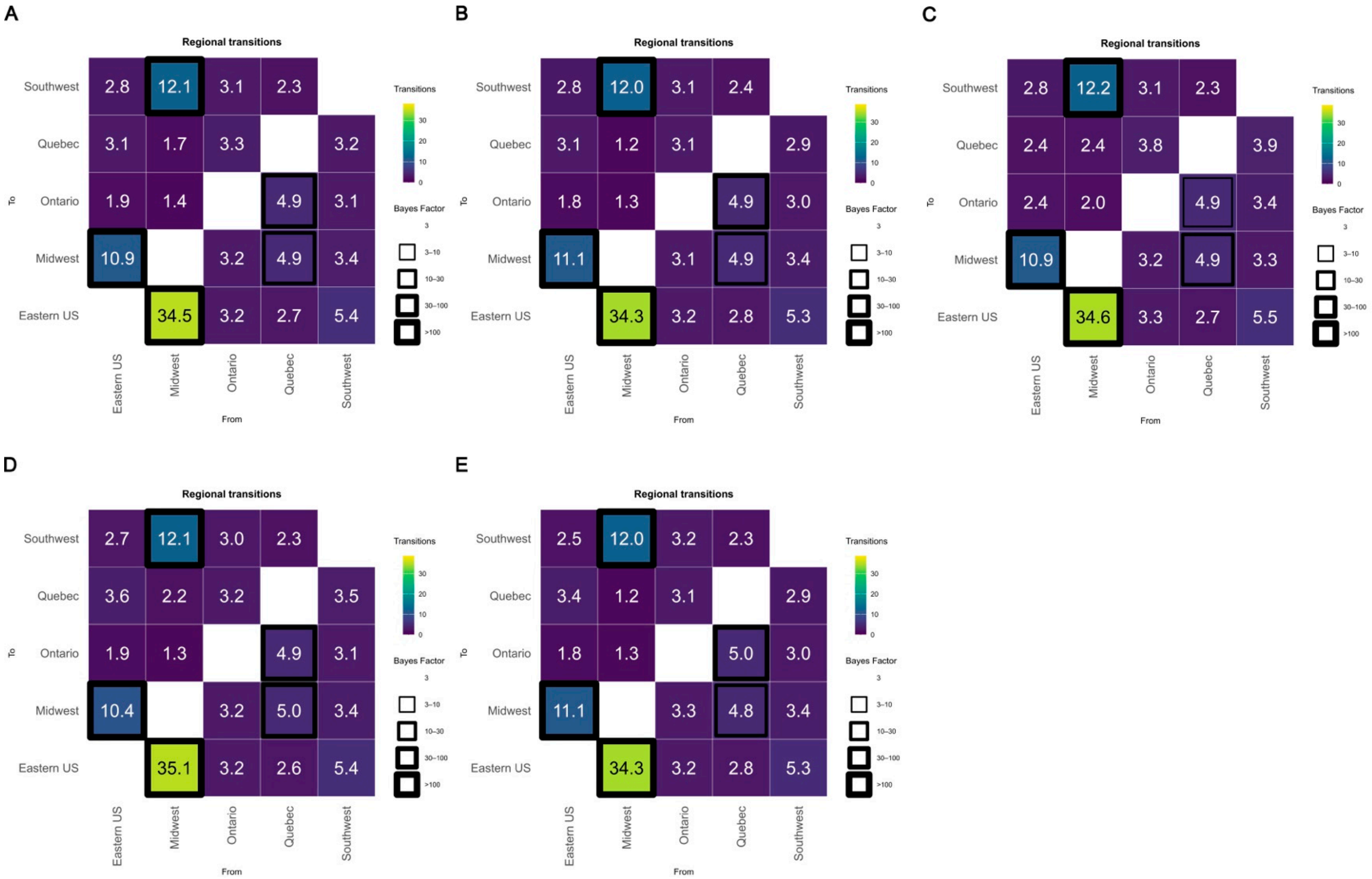

Figure S16. Spatiotemporal dynamics of the PRRSV-2 1C sub-lineage in North America. A to E) The heatmap panel on the top right displays number of transition between different regions, represented by color gradients and numerical values within each cell. Warm colors indicate high transition counts, while cool colors indicate low transitions counts. The thickness of the black boxes around the cells indicates the magnitude of the Bayes factor; only Bayes factors above 3 were considered significant.

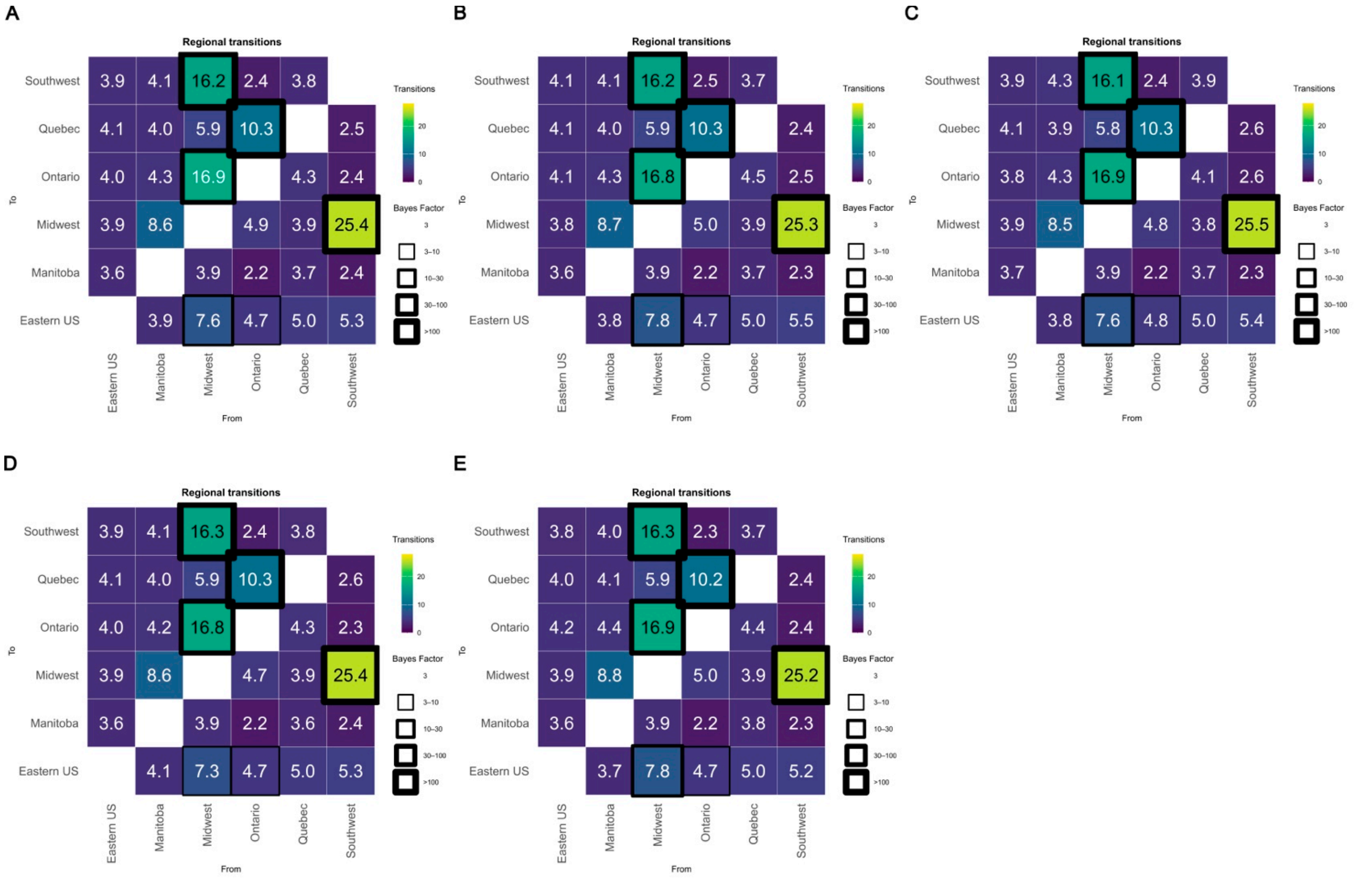

Figure S17. Spatiotemporal dynamics of the PRRSV-2 1E sub-lineage in North America. A to E) The heatmap panel on the top right displays number of transition between different regions, represented by color gradients and numerical values within each cell. Warm colors indicate high transition counts, while cool colors indicate low transitions counts. The thickness of the black boxes around the cells indicates the magnitude of the Bayes factor; only Bayes factors above 3 were considered significant.

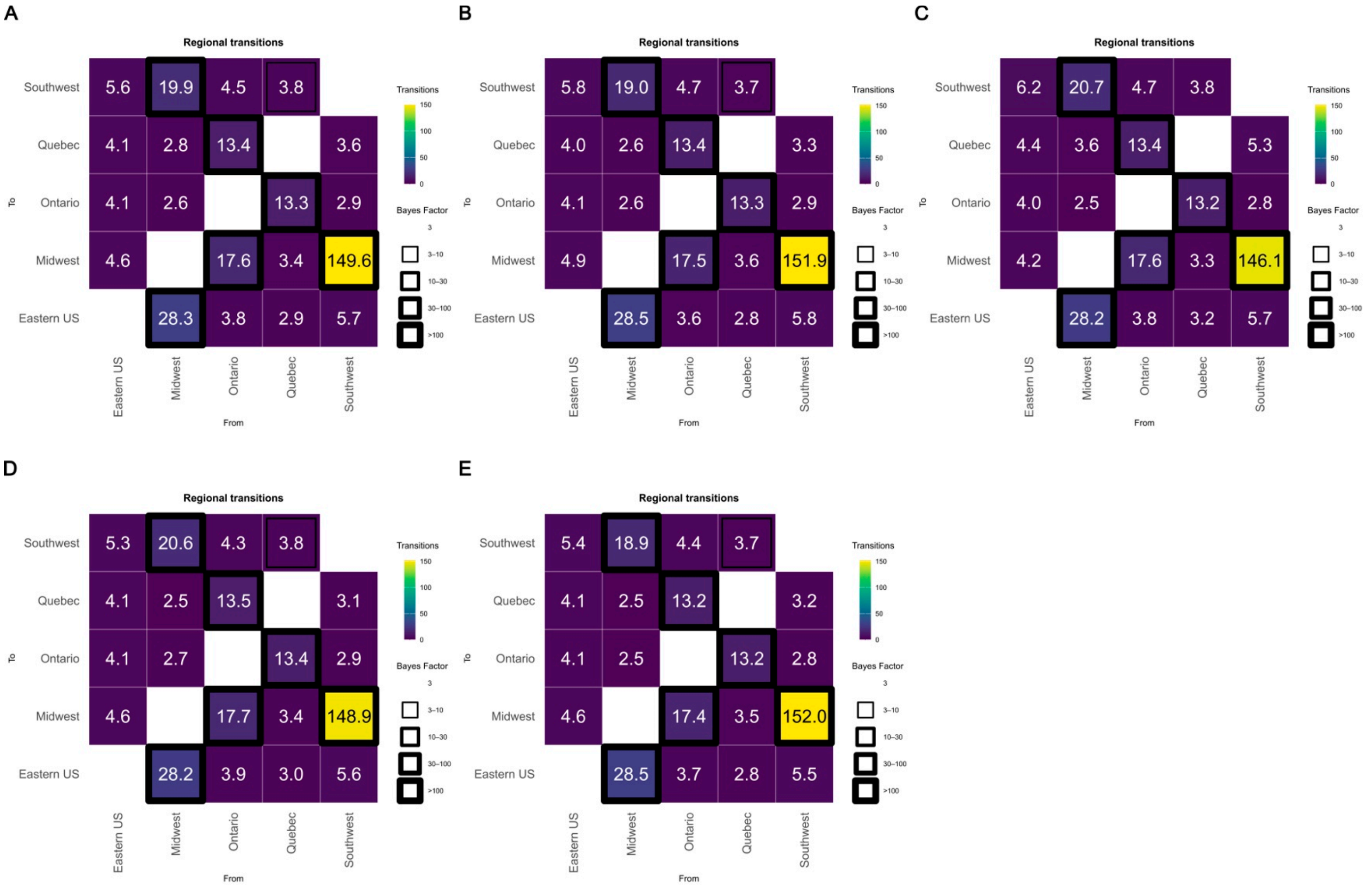

Figure S18. Spatiotemporal dynamics of the PRRSV-2 1H sub-lineage in North America. A to E) The heatmap panel on the top right displays number of transition between different regions, represented by color gradients and numerical values within each cell. Warm colors indicate high transition counts, while cool colors indicate low transitions counts. The thickness of the black boxes around the cells indicates the magnitude of the Bayes factor; only Bayes factors above 3 were considered significant.
